# Coordination of canonical and noncanonical Hedgehog signalling pathways mediated by WDR11 during primordial germ cell development

**DOI:** 10.1038/s41598-023-38017-9

**Published:** 2023-07-29

**Authors:** Jiyoung Lee, Yeonjoo Kim, Paris Ataliotis, Hyung-Goo Kim, Dae-Won Kim, Dorothy C. Bennett, Nigel A. Brown, Lawrence C. Layman, Soo-Hyun Kim

**Affiliations:** 1 Molecular and Clinical Sciences Research Institute, St. George’s, University of London, London, UK; 2 Present Address: Kernel Diagnostic Laboratories LTD, London, UK; 3 Present Address: The Babraham Institute, Cambridge, UK; 4 Institute for Medical and Biomedical Education, St. George’s, University of London, London, UK; 5 Neurological Disorders Research Center, Qatar Biomedical Research Institute, Hamad Bin Khalifa University, Doha, Qatar; 6 Department of Biochemistry, Yonsei University, Seoul, Republic of Korea; 7 Section of Reproductive Endocrinology, Infertility and Genetics, Department of Obstetrics and Gynecology, Department of Neuroscience and Regenerative Medicine, Department of Physiology, Medical College of Georgia, Augusta University, Augusta, USA

**Keywords:** Cell biology, Developmental biology, Genetics, Molecular biology, Diseases, Endocrinology, Medical research

## Abstract

*WDR11*, a gene associated with Kallmann syndrome, is important in reproductive system development but molecular understanding of its action remains incomplete. We previously reported that *Wdr11*-deficient embryos exhibit defective ciliogenesis and developmental defects associated with Hedgehog (HH) signalling. Here we demonstrate that WDR11 is required for primordial germ cell (PGC) development, regulating canonical and noncanonical HH signalling in parallel. Loss of WDR11 disrupts PGC motility and proliferation driven by the cilia-independent, PTCH2/GAS1-dependent noncanonical HH pathway. WDR11 modulates the growth of somatic cells surrounding PGCs by regulating the cilia-dependent, PTCH1/BOC-dependent canonical HH pathway. We reveal that PTCH1/BOC or PTCH2/GAS1 receptor context dictates SMO localisation inside or outside of cilia, respectively, and loss of WDR11 affects the signalling responses of SMO in both situations. We show that GAS1 is induced by PTCH2-specific HH signalling, which is lost in the absence of WDR11. We also provide evidence supporting a role for WDR11 in ciliogenesis through regulation of anterograde intraflagellar transport potentially via its interaction with IFT20. Since WDR11 is a target of noncanonical SMO signalling, WDR11 represents a novel mechanism by which noncanonical and canonical HH signals communicate and cooperate.

## Introduction

The Hedgehog (HH) signalling pathway determines cell fate and tissue patterning during development and regulates homeostasis^1,2^. Mammals have three HH ligands – Sonic, Indian and Desert – that diffuse to target cells and bind to the primary receptor, Patched (PTCH) 1 or 2. Although structurally similar, PTCH2 has shorter intracellular terminals than PTCH1^3^ and shows distinct expression patterns^4,5^. The role of PTCH2 in development remains unclear. There are three obligatory co-receptors of HH: growth arrest-specific gene 1 (GAS1), cell-adhesion-molecule-related/downregulated by oncogenes (CDO, also called CDON), and Brother of CDON (BOC). CDO/BOC are cell adhesion proteins containing immunoglobulin/fibronectin type III domains^6,7^. GAS1 localises at membrane rafts via a glycosylphosphatidylinositol anchor^8^. These co-receptors bind HH independently of PTCH, facilitating ligand-receptor interactions at the cell surface^6,9^ to regulate the activity of Smoothened (SMO), a central signal transducer. Canonical HH signal activation requires translocation of SMO to the primary cilium, which prevents the proteolytic cleavage of GLI transcription factors, resulting in GLI-driven morphogenesis. Cilia-independent and GLI-independent SMO activation also occurs^10,11^. This noncanonical HH signalling has various outcomes such as cytoskeletal rearrangement, cell migration and axon guidance^12–14^. Current understanding of noncanonical HH signalling in development remains sparse and its spatiotemporal regulatory mechanism is unknown. Growing evidence indicates that the canonical and noncanonical pathways occur in separate cellular compartments triggering distinct molecular pathways. Noncanonical signalling either promotes^15^ or antagonises^16^ canonical signalling, but how they crosstalk in vivo is unclear. We and others have suggested that the intracellular location of SMO might be the critical determinant^10,11^.

Primary cilia play important roles in development and normal physiology^17,18^. The assembly and maintenance of primary cilia depend on intraflagellar transport (IFT), a bi-directional protein trafficking system. IFT-A drives retrograde transport from the ciliary tip to the base via dynein motors, while IFT-B mediates anterograde transport from the cytoplasm to the ciliary tip^19^. IFT is essential for the correct localisation of receptors and signalling molecules within cilia during signal transduction. Extension of the ciliary axoneme requires microtubule organisation and cargo transport by the IFT complex^20^, but how the subunit proteins are recruited to the basal body (the mother centriole) during early ciliogenesis is not fully understood.

Primordial germ cells (PGCs) are the bipotent precursors of gametes. Developing PGCs undergo distinctive stages before differentiating into either spermatozoa or oocytes in the gonads^21^. In mice, PGCs originate from the posterior primitive streak (Embryonic day 7.5, E7.5) and move into the hindgut where they migrate along its anterior extension (E8-E9.5). Then they leave the hindgut, travel through the mesentery of the dorsal body wall, and finally enter the bilateral genital ridges (GR) at E10.5. PGC migration is regulated by networks of signalling molecules and receptors expressed in the germ cell niche^22,23^. Earlier studies in Drosophila and zebrafish demonstrated that HH signalling is involved in PGC development, but not as a guidance cue or fate determinant^24–26^. We previously showed that HH is not a chemoattractant for PGCs in mice, but noncanonical HH signalling via phosphorylation of CREB and Src is essential for the motility of PGCs. We also found that PGCs are naturally unciliated and express exclusively PTCH2/GAS1 receptors on their surface, while ciliated somatic cells surrounding the PGCs express PTCH1/BOC, indicating a specific partnership between the receptors^11^. Therefore, directional migration of PGCs can illuminate the connection between cilia-dependent and -independent HH signalling in development.

WDR11 belongs to a family of proteins with WD40-repeat (WDR) domains known to serve as a scaffold for protein–protein interactions^27^. WDR11 plays a role in endosome-derived vesicle trafficking to the trans-Golgi network (TGN) and tethering of AP-1-dependent cargo^28^. WDR11 was also shown to be part of the lysosomal and autophagy network^29,30^. We identified *WDR11* mutations associated with Kallmann syndrome (KS) and congenital hypogonadotrophic hypogonadism (CHH), human genetic disorders defined by delayed puberty and infertility, along with a range of developmental defects^31^. Rare variants of *WDR11* were also reported in septo-optic dysplasia, combined pituitary hormone deficiency and pituitary stalk interruption syndrome^32–34^. *Wdr11* knockout (KO) mice die at mid-gestation, but those that survived are in/sub-fertile with hypoplastic gonads containing fewer germ cells compared to the wild type (WT)^35^. To date, the role of WDR11 in PGC development has not been investigated. Wdr11 is required for ciliogenesis and *Wdr11*-deficient cells exhibit defective cilia^35^. Mutations of cilium-related genes often affect canonical HH signalling, but it is unclear whether mutations of WDR11 disrupt signalling because of defective ciliary architecture or via a more direct role in signalling.

Here we demonstrate a role for WDR11 in the proliferation and migration of PGCs and the development of gonads. WDR11 exerts multiple functions in HH signalling and connects canonical and noncanonical pathways, cooperation between which is essential for normal gonad development.

## Results

### WDR11 is expressed in the PGC developmental niche in both ciliated and unciliated cell types

*Wdr11* mRNA was expressed in the developing and adult urogenital organs of both sexes (Fig S1). Whole-mount X-gal and WDR11 direct immunofluorescence confirmed the presence of *Wdr11* mRNA and protein in the regions through which PGCs migrate, including the hindgut (HG) and the urogenital ridges at E9.5–11.5. Co-immunostaining for SSEA1, a carbohydrate antigen specifically expressed by PGCs, confirmed the expression of WDR11 in individual PGCs as well as in the surrounding somatic cells, which was absent in *Wdr11*^*–*/–^ embryos (Fig S1). Immunofluorescent staining of GR sections for ciliary protein Arl13b confirmed that the mesenchymal cells immediately surrounding the PGCs were ubiquitously ciliated in WT embryos. But *Wdr11*^–/–^ embryos showed a significant reduction in ciliation frequency and length, as expected. In contrast, PGCs remained unciliated, regardless of *Wdr11* status (Fig S2).

### Loss of WDR11 disrupts PGC migration in vivo

**Figure 1:**
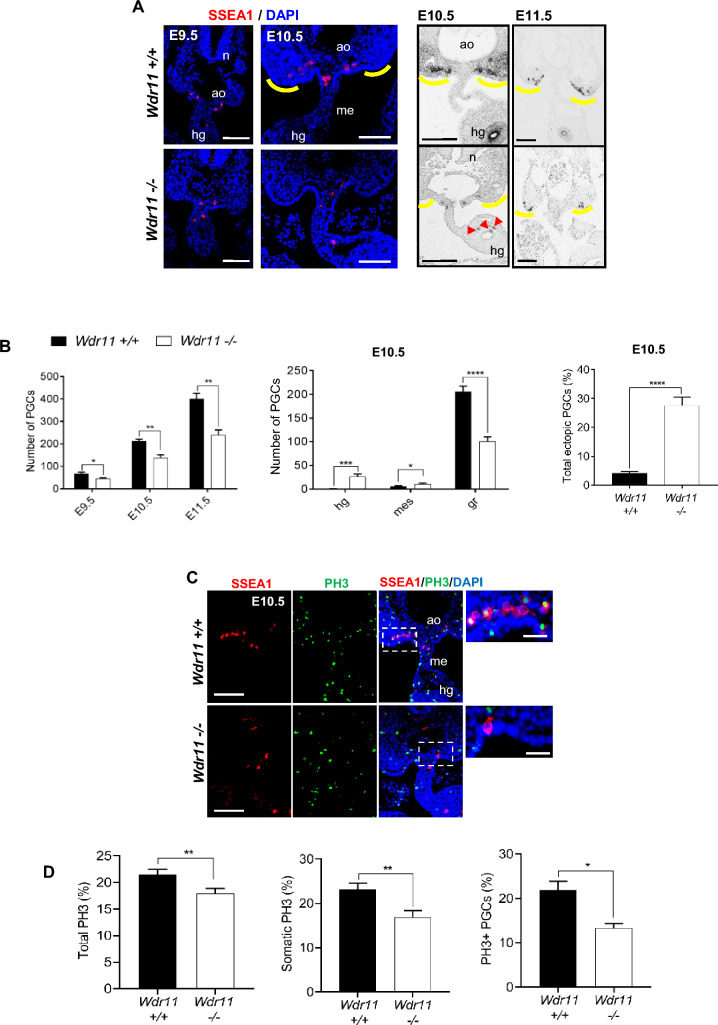
Loss of WDR11 disrupts PGC migration and proliferation. (**A**) Immunofluorescence (left) and alkaline phosphatase (right) staining of PGCs in their migratory route from the hindgut towards the GRs (yellow lines) at different developmental stages as indicated. Mis-located PGCs indicated with arrowheads. Representative images are shown. *n* neural tube, *ao* aorta, *hg* hindgut, *me* mesentery. Scale bar, 20 µm (E9.5); 100 µm (E10.5 and 11.5). (**B**) Total numbers of PGCs per embryo at each stage and location are shown for each genotype (n = 5 embryos). The total PGC number was generated by counting SSEA-positive cells from every other slide of the serial sections of E9.5, E10.5 and E11.5 embryos. The proportion of ectopic PGCs is shown as a percentage value of total PGCs at E10.5. Values are shown as mean ± SD. Unpaired Student’s *t* test (*P < 0.05; **P < 0.01; ***P < 0.001; ****P < 0.0001). *mes* mesentery, *gr* genital ridge. (**C**) Representative images of immunofluorescence staining of SSEA1 and phospho-Histone 3 (PH3) on WT and *Wdr11*^*–/–*^ GR tissue sections. Zoomed-in images of the dotted area are shown on the right. Scale bar, 100 µm (left); 20 µm (right). (**D**) Percentage of PH3-positive cells in total (left), somatic cell population (middle) and PGC population (right) are compared between WT and *Wdr11*^*–/–*^ embryos. Error bars represent SEM. Statistical analysis by unpaired Student’s *t* test (n = 7 embryos per genotype; *P < 0.05; **P < 0.01).

*Wdr11*^*–*/–^ mice are infertile. *Wdr11-*deficient testes are smaller in size and contain fewer spermatocytes and spermatids with abnormal morphology. *Wdr11*-deficient ovaries are also smaller than WT and present with disproportionally higher numbers of oogonia or primordial follicles and reduced numbers of mature follicles (Fig S3). These observations suggest that loss of *Wdr11* results in defective development of germ cells and gonads in both sexes. Since defective migration of PGCs can result in insufficient numbers of germ cells present in the gonads at birth, leading to infertility or sub-fertility and premature ovarian failure, we investigated if WDR11 participated in PGC development. First, we analysed the number and location of PGCs by anti-SSEA1 immunofluorescence and alkaline phosphatase staining. The results showed that *Wdr11*^*–*/–^ embryos were still populated with PGCs in their normal migratory path between the HG and GR at E9.5–11.5, but many PGCs were inappropriately located for the stage of development (Fig. 1A). Counts of SSEA1-positive cells confirmed significantly fewer total PGCs in *Wdr11*^*–*/–^ than in WT. Notably, PGCs accumulated inappropriately in HG and mesentery, with significantly fewer arriving in the GR at E10.5 in *Wdr11*^*–*/–^ embryos (Fig. 1B). Thus, loss of WDR11 did not prevent the specification of PGCs, but impaired migration within their normal path. Those mis-localised PGCs are unlikely to develop properly owing to the lack of necessary signals from their environment.

### *Wdr11*-deficient PGCs show reduced intrinsic motility

**Figure 2:**
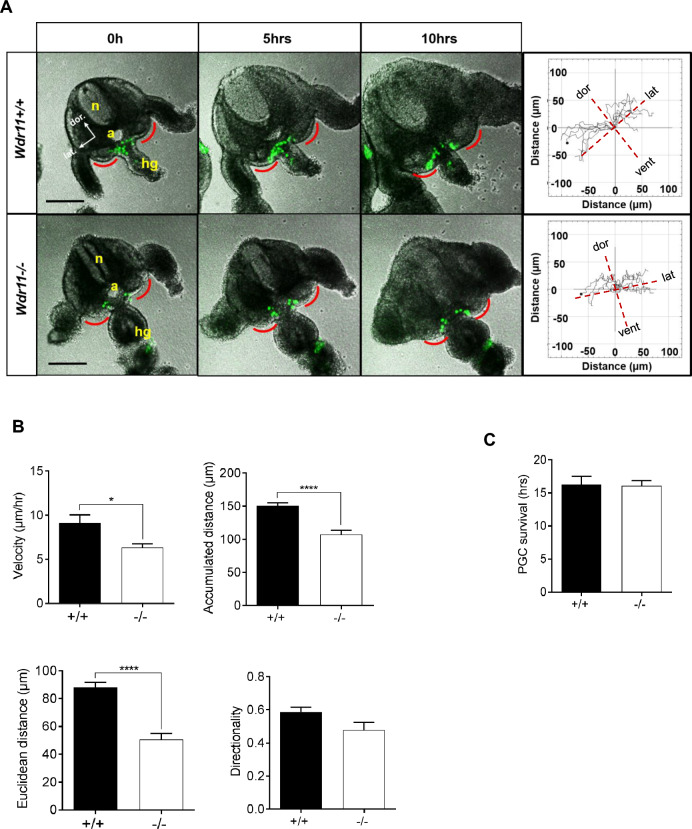
WDR11 KO disrupts PGC migration but not directionality. (**A**) Representative images of the z-stack plane at 0, 5 and 10 h from time-lapse imaging of embryo slice cultures from *Stella*^*GFP*+*/*+^*;Wdr11*^+*/*+^ and *Stella*^*GFP*+*/*+^*;Wdr11*^*–/–*^ embryos in biologically independent experiments (Supplementary Movies 1 and 2). The GFP-positive PGCs migrating towards the GRs (red line) were tracked. Trajectory plots of migration (right panel) were generated by placing the origins of all tracks at the 0,0 XY coordinate. The direction of migration in relevance to the embryo orientation is shown with dotted lines. Dor, dorsal; lat, lateral; vent, ventral. Scale bar, 100 µm. (**B**,**C**) Comparison of velocity, accumulated distance, Euclidean distance, directionality and survival of the migrating PGCs of WT and *Wdr11*^*–/–*^ embryos in the live imaging shown in (**A**), measured as described in Methods. Values are mean ± SEM from independent slice cultures (n = 9 WT; n = 6 KO) where 7–10 PGCs were tracked from each slice. Unpaired *t* test (*P < 0.05; ****P < 0.0001; directionality, *P* = 0.061; survival *P* = 0.64).

Since the specification and differentiation of PGCs appear mostly normal in *Wdr11*^*–*/–^ embryos, WDR11 may affect PGC development through one of the following processes: motility, proliferation or survival. First, we examined the motile behaviour of PGCs in WT and *Wdr11*^*–*/–^ embryos using the *Stella*^*GFP*^ hybrid line which expressed green fluorescent protein (GFP) driven by the *Stella* gene promoter. *Stella* (*Dppa3*) is the most specific marker for PGCs, being expressed soon after their specification at ~ E7.5 and maintained until E13.5 in females and E15.5 in males. We performed time-lapse live imaging of embryo slice cultures of explanted GR. The movements of GFP-labelled PGCs were tracked and analysed for directionality, targeting, distance and speed over > 10 h. We found that PGCs in both WT and *Wdr11*^*–*/–^ embryos were moving towards the GR area, showing no discernible differences in their targeting of migration (Fig. 2A). Next, we performed quantitative motion analyses, which revealed that the velocity, accumulated distance and Euclidean distance of migration were significantly reduced in *Wdr11*^*–*/–^ embryos. However, the directionality was not altered (Fig. 2B). Hence, most PGCs were still moving towards the correct destination, but the speed and distance of migration were decreased by WDR11 loss, resulting in fewer PGCs arriving at the GR. The live imaging showed some PGCs disintegrating progressively, a hallmark of apoptotic cells (Supplementary Movies 1 and 2). To ascertain whether the reduced PGC movement was due to increased cell death, we assessed the survival times by measuring the mean number of hours that the green fluorescence of individual cells could be detected in the movies and found no difference between the genotypes (Fig. 2C). These data suggest that WDR11 facilitates PGC motility during migration towards the GRs but not their targeting and attraction towards GRs, nor their survival.

### Defective proliferation but normal apoptosis in WDR11 mutants

One possible explanation for the decreased number of PGCs is reduced proliferation. In mice, PGCs continue to proliferate during and after migration, rapidly expanding to a final population of ~ 25,000 cells per embryo at E13.5. Indeed, PGCs visibly divided during time-lapse imaging (Supplementary Movies 1 and 2). To determine if loss of WDR11 affects PGC proliferation, we counted mitotically active cells by phosphorylated-histone H3 (PH3) immunostaining of GR sections (Fig. 1C). The total number of proliferating PGCs (PH3 + , SSEA1 + , DAPI +) and somatic cells (PH3 + , SSEA1–, DAPI +) between the forelimb and hindlimb buds were manually counted and PH3-labelling indices were generated against the total DAPI-positive counts. *Wdr11*^*–*/–^ embryos showed a lower mitotic index compared to WT in both PGCs and somatic cells (Fig. 1D). Therefore, WDR11 promotes the proliferation of both PGCs and the mesenchyme.

During PGC migration, there is also an up-regulation of factors involved in apoptosis, and embryos with a defective apoptotic pathway exhibited ectopic PGCs that were not cleared effectively^36^. To determine if loss of WDR11 affected apoptosis, we carried out immunostaining for cleaved caspase 3 and manually counted CASP3-positive cells against total DAPI-positive cells (Fig S4A). The results indicated a marked increase of total apoptotic cells in *Wdr11*^*–*/–^ embryos (Fig S4B, top left). However, this is likely due to increased ectopic PGCs present in these embryos (Fig S4B, bottom left), rather than enhanced apoptosis in general. This conclusion was based on the fact that the apoptotic index in the mesenchymal somatic cells was not different between the genotypes (Fig S4B, top right) and the ectopic PGCs were similarly positive for CASP3 in WT and Wdr11^*–*/–^ (Fig S4B, bottom right). Therefore, loss of WDR11 did not cause an overall increase in apoptosis, confirming our observation from the time-lapse imaging (Fig. 2C).

### Expression of PGC developmental genes is mostly unaffected in WDR11 mutants

Several genes and their signalling pathways are known to be important in PGC development, such as *Blimp1, c-Kit (Kit), Steel (Kitl), Cxcr4* and *Sdf1 (Cxcl12)*^22^. We examined if WDR11 loss affected the expression of any of these genes. Initial screening confirmed their expression in the PGC migratory niche and adult urogenital organs (Fig S1B). Quantitative analyses of dissected GR tissue indicated that WDR11 loss did not reduce mRNA levels of these regulators, except for *c-Kit*. There was also a numerical but non-significant reduction in *Cxcr4* (Fig S5). However, this is likely due to the reduced number of PGCs in the mutants, as both *c-Kit* and *Cxcr4* are cell surface receptors expressed by PGCs but not GR mesenchyme^37,38^. The expression of the respective ligands for these receptors, *Steel* and *Sdf1*, which mediate the chemo-attraction of PGCs towards the gonads, was not altered (Fig S5). Since the reduction in *c-Kit* mRNA alone cannot fully explain the reduced proliferation and migration of PGCs in *Wdr11*^-/-^ embryos, additional mechanisms must be involved. PGCs and their surrounding somatic cells originate from different embryonic lineages and PGCs migrate mainly guided by their interactions with the niche environment, so we sought to define the WDR11-mediated molecular events in these cellular contexts and especially whether canonical or noncanonical HH signalling is involved. The fact that WDR11 is expressed both in the ciliated mesenchyme and the unciliated PGCs made us also question if WDR11 has a same or different role in the two cell types.

### WDR11 mutations affect somatic cell proliferation

Our analysis of PH3 staining of GR tissue sections indicated a significant reduction in the somatic cell proliferation in *Wdr11*-null embryos (Fig. 1C,D). To further validate this finding and determine the effects of clinically identified WDR11 mutations, we employed NIH3T3, a mouse embryo fibroblast line, as a somatic cell model. We generated a panel of NIH3T3/Cas9 engineered to express mouse homologues of different disease-associated mutations of *WDR11*. The MT variant (p.Pro537Leu in human) was originally found in 2 brothers with KS/CHH presenting delayed puberty and childhood obesity^35^. The RC variant (p.Trp595Arg in human) was found in a 61-year old male patient with high grade clear-cell renal cell carcinoma^39^. For comparison, we also generated a targeted deletion of *Ift88*, a well-established ciliary gene^40^. The specific mutations and targeted KO were confirmed by Sanger sequencing of genomic DNA and Western blotting of the endogenous proteins (Fig S6A). Cell counts in growth medium over 3 days showed that targeted *Wdr11* KO severely attenuated NIH3T3 cell proliferation compared to control cells transfected with Cas9/gRNA empty vector (Fig S7A). Interestingly, cells expressing the *Wdr11*-MT variant showed moderate growth inhibition, consistent with mild loss of function as previously reported^35^. In contrast, the *Wdr11*-RC variant showed enhanced cell proliferation, consistent with a gain-of-function and pro-mitogenic effect associated with malignancy (Fig S7A). These data confirmed a correlation between WDR11 mutations and somatic cell proliferation, potentially explaining the small size and defective morphogenesis in *Wdr11*^-/-^ gonads (Fig S3).

### WDR11 KO disrupts canonical HH signalling in somatic cells

Since HH pathway genes are expressed in the mesenchyme of the PGC migratory niche^11^ and HH is known to regulate the proliferation of different cell types, we examined the role of WDR11 in canonical HH signalling represented by the transcriptional induction of target genes. *Ptch1* and *Gli1/2/3* mRNA levels were upregulated in WT GR tissue from E9.5, reaching a maximum at E10.5 followed by a gradual decrease till E12.5 (Fig S8A), suggesting active canonical HH signalling. However, *Wdr11*^*–*/–^ GR tissue failed to induce these genes even at the E10.5 peak (Fig S8B). *Boc*, but not *Cdo*, is broadly expressed in the PGC migratory niche^11^. *Wdr11*^*–*/–^ GR expressed significantly reduced levels of *Boc* mRNA compared to WT (Fig S8C). Since SHH regulates PGC motility^11^ and Desert Hedgehog (DHH) is involved in genitourinary tract development^41^, we next investigated if the expression of *Shh* or *Dhh* was altered in *Wdr11*^*–*/–^. Our qRT-PCR data of WT embryos showed *Dhh* was indeed expressed in the GR tissues, at a slightly higher level than *Shh*, but there was no significant change during E9.5–E12.5 (Fig S8D). Notably, loss of WDR11 did not affect the expression of *Dhh* or *Shh* (Fig S8E). Therefore, defective canonical HH signalling in *Wdr11*^*–*/–^ mesenchyme was not due to reduced expression of HH ligands but rather to defective reception of ligands, associated with reduced expression of PTCH1 and BOC.

### WDR11 mutations affect the ciliation of somatic cells

Given the importance of primary cilia in canonical HH signalling, we assessed the effects of various WDR11 mutations in ciliogenesis by examining the NIH3T3/Cas9 cell panel. There was no significant alteration in gross cell morphology or general cytoskeletal architecture (Fig S6B), but when we induced ciliogenesis by serum starvation, *Wdr11* KO and the *Wdr11*-MT mutant showed significantly shorter cilia compared to WT. *Ift*88 KO, employed as a positive control, also inhibited cilium formation, as expected. Interestingly, the *Wdr11*-RC mutation had only a marginal effect (Fig S7B). These data demonstrate that WDR11 is important for somatic cell ciliation and that disease-associated mutations of WDR11 affect ciliogenesis.

### WDR11 regulates IFT-B by interacting with the IFT20 complex

**Figure 3:**
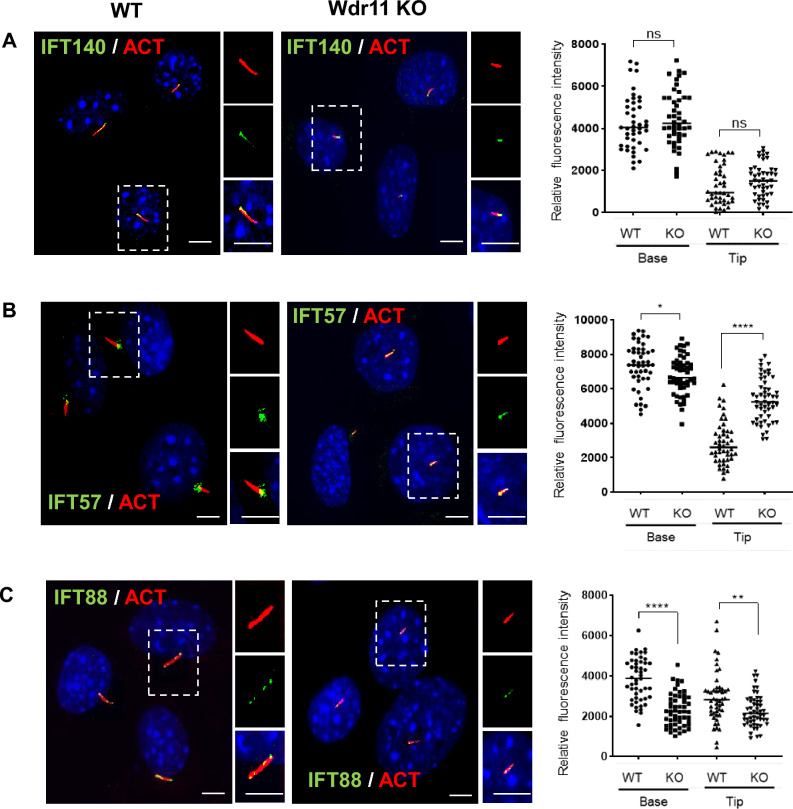
WDR11 regulates IFT-B. (**A**) Immunofluorescence analysis of IFT140 in WT and *Wdr11* KO NIH3T3/Cas9 cells co-stained with the axoneme marker, acetylated tubulin. Quantification of IFT140 localisation at the base or the tip of cilia is shown on the right (n = 86 WT; n = 92 KO). (**B**) Localisation analyses of IFT57 in WT and *Wdr11* KO cells with quantification (n = 96 WT; n = 104 KO). (**C**) Localisation analysis of IFT88 in WT and *Wdr11* KO cells with quantification (n = 98 WT; n = 106 KO). Unpaired *t* test with Welch’s correction (*P = 0.0716, **P = 0.0019, ****P < 0.0001). Scale bar, 5 µm.

To further determine the role of WDR11 in ciliogenesis, we broadly examined the structure of *Wdr11*^*–*/–^ cilia by transmission electron microscopy. *Wdr11*-deficient tissues were infrequently ciliated compared to the WT, with numerous basal bodies which failed to extend the axoneme. Those cilia that managed to extend the axoneme were much shorter. In cross-sections, *Wdr11*^*–*/–^ cilia showed a reasonably normal-appearing microtubule structure without any accumulation of vesicular particles (Fig S2D). Molecular markers for cilia such as Arl13b and acetylated-tubulin were still detectable in *Wdr11*^*–*/–^ cilia (Fig S2), hence the key structural axoneme proteins seemed to be expressed normally. It is reported that cilia with defective IFT-A exhibit normal length but form bulges at the tip with accumulation of intraciliary vesicles, while IFT-B mutants exhibit short or absent cilia^42^. Since the morphology of WDR11-defective cilia resembles the latter, we contemplate that WDR11 regulates IFT-B trafficking. So, we examined the effects of *Wdr11* KO in ciliary localisation of representative subunits of IFT-A (IFT140) and IFT-B (IFT57, IFT88). Immunofluorescence analyses of NIH3T3/Cas9 revealed a similar distribution pattern of IFT140 in WT and *Wdr11* KO, indicating normal IFT-A. But the distribution of IFT57 and IFT88 were different in *Wdr11* KO cells compared to WT, not only at the tip but also near the base of the cilium (Fig. 3). These results demonstrate that the loss of WDR11 disturbed IFT-B movement, resulting in abnormal localisation of IFT-B particles, but the effects on the core IFT-B protein (IFT88) and the peripheral IFT-B protein (IFT57) might be different.

**Figure 4:**
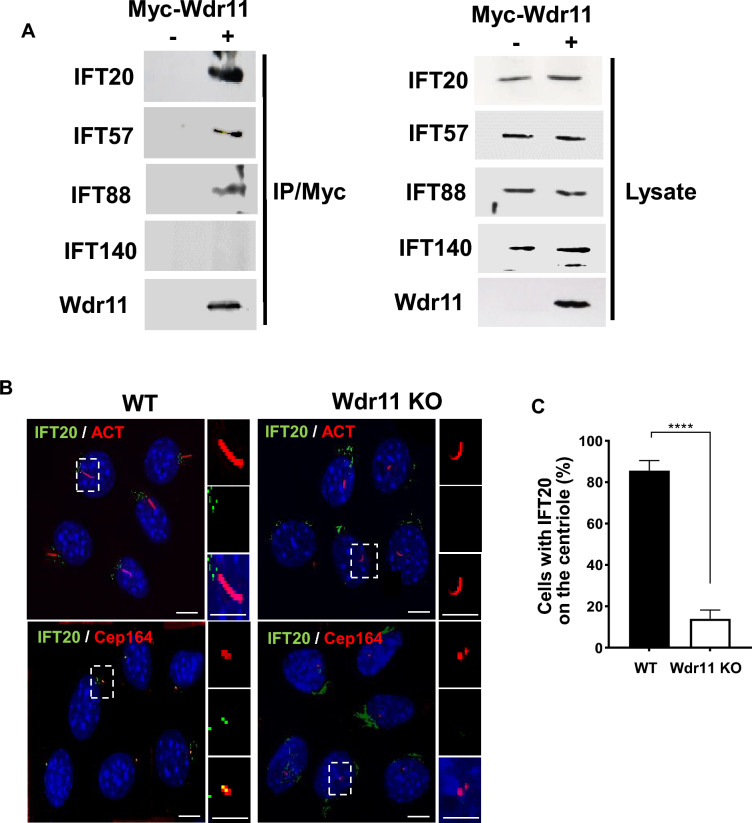
WDR11 interacts with IFT20. (**A**) NIH3T3 cells transfected with Myc-tagged WDR11 or empty vector were analysed by Western blot using respective antibodies as indicated after immunoprecipitation with anti-Myc antibody. (**B**) Localisation of IFT20 in WT and *Wdr11* KO NIH3T3/Cas9 cells co-stained with the acetylated tubulin (for axoneme) and Cep164 (for basal body/centriole). Scale bar, 5 µm. (**C**) Comparison of the number of cells with IFT20 located on the centriole counted from 11 random fields of cells (n = 117 WT; n = 128 KO). Unpaired *t* test with Welch’s correction (****P < 0.0001).

IFT20 is a major component of IFT-B and forms a complex with IFT52, IFT57 and IFT88, but not IFT140^43^. Also, IFT20 is the only IFT component shown to localise to the TGN and transport of IFT20 between the Golgi and the centrosome at the base of a cilium is essential for ciliogenesis^43,44^. We speculate WDR11 may interact with IFT20 and its binding partners in the IFT-B complex. Co-immunoprecipitation using NIH3T3 cells transfected with Myc-tagged WDR11 or empty vector demonstrated that WDR11 can form a complex with IFT20, IFT57 and IFT88, but not with IFT140 (Fig. 4A), confirming a specific interaction of WDR11 with IFT-B particles. We next asked if WDR11 is involved in ciliary trafficking of IFT20. Golgi stacks closely opposed to the basal body and IFT20 localises to both the Golgi and the mother centriole during early stage of ciliogenesis, after being recruited from the Golgi to the centriole. Our colocalisation study of IFT20 with a centriole marker Cep164 and the quantitative analysis of the positioning of IFT20 in relation to the centriole demonstrated that in the absence of WDR11, the normal compact perinuclear ribbon-like localisation of IFT20 close to a cilium base is disrupted, giving loosely dispersed and disoriented clusters away from the centrioles (Fig. 4B,C). Therefore, localisation of IFT20 to the Golgi may still be maintained but its targeted movement to the peri-basal body pool at the base of the cilium is markedly reduced by *Wdr11* KO. These data suggest that without WDR11, IFT20 cannot form efficient connections to the basal body, essential for the assembly and transport of ITF-B proteins required for axoneme extension, potentially explaining the short and infrequent cilia in Wdr11^*–*/–^ mesenchyme (Fig S2).

### WDR11 KO disrupts noncanonical HH signalling in PGCs

**Figure 5:**
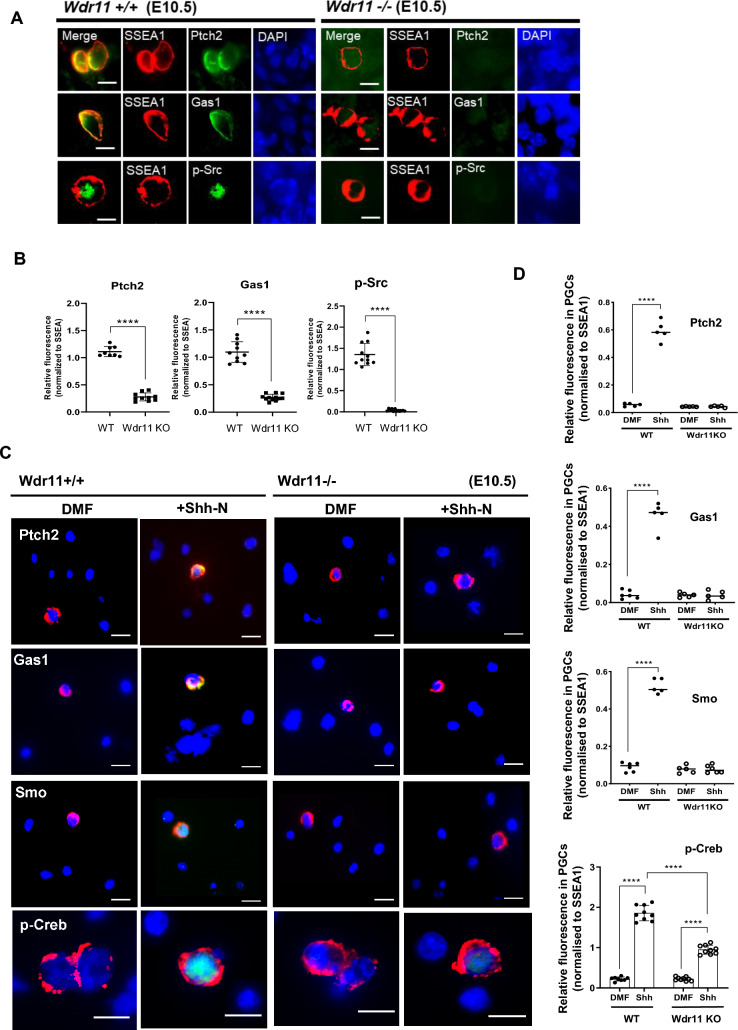
Defective noncanonical HH signalling in *Wdr11*^–/–^ PGCs. (**A**) Representative images of immunofluorescence of PTCH2, GAS1 and p-Src on the WT and *Wdr11*^*–/–*^ GR sections. The merged images are shown without DAPI signal to improve the clarity. Scale bar, 10 µm. (**B**) The relative fluorescence intensity values were normalised with those of SSEA1 in each cell as in (**A**), since the expression of SSEA1 was not itself impacted by the *Wdr11* KO. Data for PTCH2 (n = 8 WT; n = 10 KO), GAS1 (n = 10 WT; n = 12 KO) and p-Src (n = 11 WT; n = 12 KO) represented as means ± SD after unpaired *t* test with Welch’s correction (****P < 0.0001). (**C**) PGCs (labelled by SSEA1, red) in the GR primary cultures of WT and *Wdr11*^*–/–*^ were analysed by the respective antibodies as indicated after the addition of solvent dimethylformamide (DMF) or recombinant SHH protein (Shh-N) for 10 min. Representative images with merged signals (yellow) are shown with DAPI (blue). Scale bar, 10 µm. (**D**) The normalised relative intensity values of respective proteins in each cell as in (**C**) were compared for WT (DMF n = 8; Shh-N n = 9) and KO (DMF n = 9; Shh-N n = 9). Values are means ± SD after unpaired *t* test with Welch’s correction (****P < 0.0001).

Loss of primary cilia cannot explain the defective proliferation and migration of *Wdr11*^*–*/–^ PGCs because PGCs respond to HH signalling via cilia-independent mechanisms. We have previously shown that the naturally unciliated PGCs express PTCH2/GAS1 on their surface, which cooperatively receive HH. Upon ligand binding, the PTCH2/GAS1 hetero-complex mediates the rapid derepression of SMO, inducing p-Src and p-CREB. Activation of this noncanonical HH signalling in PGCs coincided with an increased accumulation of SMO in the cytoplasm and plasma membrane^11^. To define the role of WDR11 in the context of PGCs, we examined the status of PTCH2 and GAS1 in WT and *Wdr11*^-/-^ GR sections. Immunofluorescence analyses showed that the expression of PTCH2 and GAS1 was virtually abolished in *Wdr11*^*–*/–^ PGCs, suggesting defective responsiveness to HH due to a lack of appropriate receptors. Accordingly, there was impaired phosphorylation of Src, a regulator of motility and proliferation in migrating PGCs (Fig. 5A,B). To further assess the signalling capacity of *Wdr11*^*–*/–^ PGCs, we generated primary GR cultures, stimulated them with recombinant SHH (Shh-N) and measured the responses of HH pathway components by quantitative immunofluorescence. Upon SHH treatment, normalised intensities of PTCH2 and GAS1 were significantly increased in WT PGCs, indicating that they are targets of HH signalling. However, *Wdr11*^*–*/–^ PGCs showed no induction of these receptors. The normalised intensity of SMO was also increased by SHH in WT, but not in *Wdr11*^*–*/–^ PGCs (Fig. 5C,D). PTCH2/GAS1-mediated noncanonical HH signalling elicits a global activation of cAMP signalling, inducing p-CREB in the cytoplasm^11^. Since activation of CREB has a pivotal role in cell proliferation and motility and agents that increase intracellular cAMP levels such as forskolin enhanced PGC proliferation^45^, we tested whether *Wdr11*^*–*/–^ PGCs were able to induce p-CREB in response to HH. Primary GR cultures serum-starved for 24 h exhibited little basal p-CREB. SHH treatment markedly increased p-CREB in WT, but significantly less in *Wdr11*^*–*/–^ PGCs (Fig. 5C,D and Fig S9A). GLI3 was hardly detectable in PGCs, showing no changes upon HH (Fig S9B). Therefore, PTCH2/GAS1-dependent noncanonical HH signalling mediated by downstream effectors SMO, Src and CREB was largely abolished in *Wdr11*^*–*/–^ PGCs, potentially underlying their defective migration and proliferation (Figs. 1 and 2).

### GAS1 is a specific target of SHH/PTCH2 signalling

**Figure 6:**
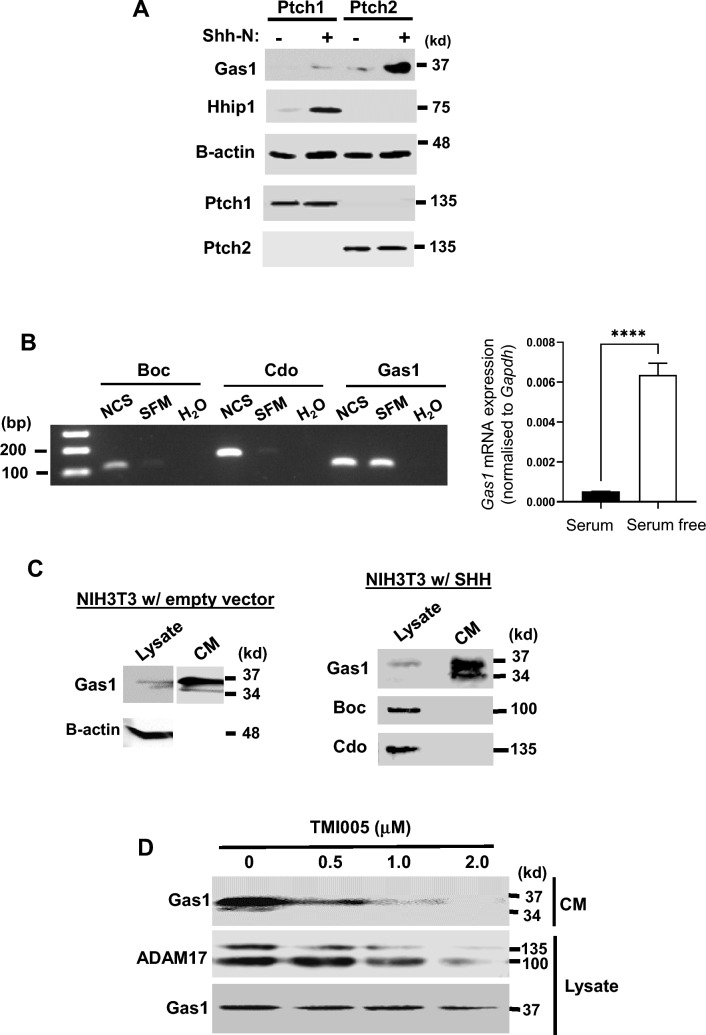
PTCH2/SHH signalling induces GAS1 expression. (**A**) NIH3T3/Cas9 cells expressing PTCH1 or PTCH2 individually were treated with ( +) or without (–) Shh-N and analysed by Western blotting for the endogenous proteins as indicated. B-actin as a loading control. (**B**) The mRNA levels of *Boc, Cdo* and *Gas1* in WT NIH3T3 cells cultured in either 10% newborn calf serum-containing medium (NCS) or 0.5% serum-containing medium (SFM) for 24 h were analysed by RT-PCR and agarose gel electrophoresis. H_2_O was used as the template in the negative control (Left panel). *Gas1* mRNA levels in WT NIH3T3 cultured in NCS or SFM were quantitatively assessed by real-time qRT-PCR (n = 3; unpaired student’s *t* test, P = 0.000066) (Right panel). (**C**) Western blot analyses of the cell lysate and the conditioned medium (CM) collected from WT NIH3T3 cells which were transfected with either an empty vector or SHH expression construct and cultured in SFM for 48 h. The full-length (37 kd) and the cleaved form (34 kd) of GAS1 protein are detected in CM. (**D**) Western blot analyses of the CM or cell lysates of NIH3T3 cells treated with varying concentrations of TMI005 (ADAM17 specific inhibitor) as indicated, for 48 h in SFM. The pro-form (135kd) and the mature form (100kd) of ADAM17 are detected in the lysate.

We previously showed that WDR11 is a target of SMO-dependent noncanonical HH signalling. SMO agonist increased *Wdr11* mRNA, but not via GLI^35^. The observation that SHH substantially induced GAS1 protein in PTCH2-expressing PGCs, but not in PTCH1-expressing somatic cells (Fig. 5C) made us wonder if GAS1 is also a downstream target of noncanonical signalling. To investigate this, we utilised NIH3T3/Cas9 cell lines engineered to express PTCH1 or PTCH2 individually in a *Ptch1/2* double-null background, a unique model to explore distinct HH-driven signalling activities. Of note, NIH3T3 cells express endogenous GAS1, BOC and CDO^11^. PTCH2-expressing NIH3T3/Cas9 cells stimulated with SHH, showed a clear upregulation of GAS1. PTCH1-expressing cells, however, barely induced GAS1, if at all. To validate the system, we also examined in parallel the expression of HHIP1, a well-known target of SHH/PTCH1 signalling^4^, and found that HHIP1 was prominently induced only in PTCH1-expressing cells (Fig. 6A). These results support the notion that GAS1 is a specific target of SHH/PTCH2 signalling and explain the failed GAS1 expression in *Wdr11*^-/-^ PGCs which lack PTCH2 (Fig. 5).

To understand how GAS1 expression is regulated, we examined its transcription. GAS1 was originally identified as one of the highly induced mRNAs in serum-starved NIH3T3 during a screen to identify mammalian growth inhibitors^46,47^. We indeed found that *Gas1* mRNA was increased by 50 fold upon serum withdrawal in WT NIH3T3 cells (Fig. 6B, right panel). On the contrary, the transcription of *Boc* and *Cdo* was turned off in this condition (Fig. 6B, left panel). Since WT NIH3T3 expresses PTCH1 endogenously and the serum-free medium was used to treat cells with or without SHH in our experimental setting, the failed induction of GAS1 protein in PTCH1 context could not be explained at the transcriptional level. GAS1 is a GPI-anchored protein localised to the cholesterol-rich lipid rafts known to play a regulatory role by ectodomain shedding of various surface proteins^48,49^^.^ The existence of secretory form of GAS1 has also been reported^50–52^. Therefore, we hypothesised that there might be a negative regulation of GAS1 by protein shedding. To test this idea, we examined GAS1 in the conditioned medium of WT NIH3T3 culture by Western blotting and found that a high level of GAS1 was present in the conditioned medium, while a relatively low level of GAS1 was detected in the cell lysate. We also observed an additional band of GAS1 appearing in the conditioned medium, likely a product after the cleavage of GPI anchor. In contrast, BOC and CDO were only present in the cell lysate with no evidence of shedding (Fig. 6C). The disintegrins and metalloproteinases (ADAM) 10 and 17 were reported to mediate the shedding of various GPI-anchored proteins^53^ and Shh-Np^54^. We found that treatment with ADAM17 inhibitor TMI0005 caused a concentration-dependent inhibition on GAS1 shedding in NIH3T3 (Fig. 6D). These results suggest a possibility of a posttranslational regulation of GAS1 via the extracellular protease-dependent shedding and ADAM17 may play a role in this process.

### PTCH1 and PTCH2 govern SMO localisation in relation to primary cilia

**Figure 7:**
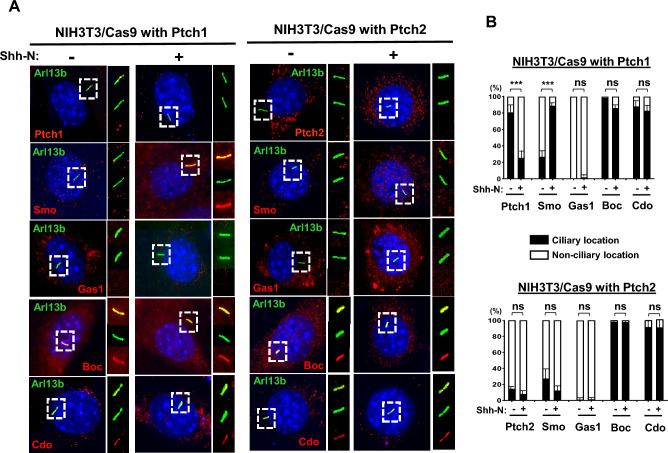
PTCH1 and PTCH2 dictate the differential localisation of SMO. (**A**) NIH3T3/Cas9 cells expressing PTCH1 or PTCH2 individually were treated with or without Shh-N to determine the intracellular location of the respective proteins (red) in relevance to primary cilia (labelled with Arl13b, green). (**B**) Cells with ciliary co-localisation (yellow in the merged images shown in the top panel of (**A**)) in each protein/treatment group are scored by counting 50 ~ 100 cells from 5 random fields. Data presented as mean ± SD with unpaired *t* test (***P < 0.001; ns, not significant).

Noncanonical signalling is a cilia-independent event that can occur in ciliated cells, in parallel with canonical signalling. Also, it occurs quite rapidly, within minutes, while the canonical activation of GLI takes > 24 h to peak^11^. We hypothesised that the specific PTCH and co-receptor context may dictate the location of SMO, thus the mode of its signalling activity. To test this notion, we investigated the location of these molecules before and after SHH stimulation in NIH3T3/Cas9 cells expressing PTCH1 or PTCH2, respectively. Immunofluorescence data showed that in the basal state, PTCH1 was mostly associated with primary cilia, while a weak punctate SMO signal was distributed in the cytoplasm. The addition of SHH caused the exit of PTCH1 from the cilia, with concomitant entry of SMO into cilia, as expected (Fig. 7). In PTCH2-expressing cells, however, both PTCH2 and SMO remained mostly outside the cilia, irrespective of SHH treatment. Therefore, SMO did not efficiently translocate to the cilia in the PTCH2 context. Interestingly, GAS1 was excluded from cilia, while BOC and CDO remained within cilia in both PTCH1- and PTCH2-expressing cells regardless of SHH stimulation (Fig. 7). Our previous biochemical studies demonstrated that PTCH1 constitutively binds BOC but, upon Shh-N addition, BOC gradually transfers the ligand to PTCH1, slowly causing separation of PTCH1 from BOC, which coincides with full activation of SMO-dependent canonical signalling^11^. Combined with the current data, it supports a model where BOC/CDO serves as the ciliary adhesion site for PTCH1 in the resting state, but upon SHH binding, PTCH1 dissociates from BOC/CDO, resulting in its expulsion from cilia. In contrast, the interaction of SMO and PTCH2/GAS1 occurs mainly outside of cilia, and ciliary translocation of SMO does not occur (or occurs ineffectively) even after SHH addition. Therefore, in the context of PTCH2/GAS1, SMO may function like a G protein-coupled receptor (GPCR) in the cytoplasm, leading to the rapid activation of downstream signalling as evident in PGCs. *Wdr11*^*–*/–*-*^ soma with defective cilia showed defective expression and signalling of these ciliary receptors (PTCH1, BOC, SMO) as shown in Fig. S8. Intriguingly, the expression and responses of non-ciliary receptors (PTCH2, GAS1, SMO) were also affected in *Wdr11*^*–*/–^ PGCs (Fig. 5). These results suggest a role for WDR11 in the regulation of HH signal pathways, both inside and outside of the cilia.

### Somatic mutations of WDR11 affect PGC proliferation in co-culture

**Figure 8:**
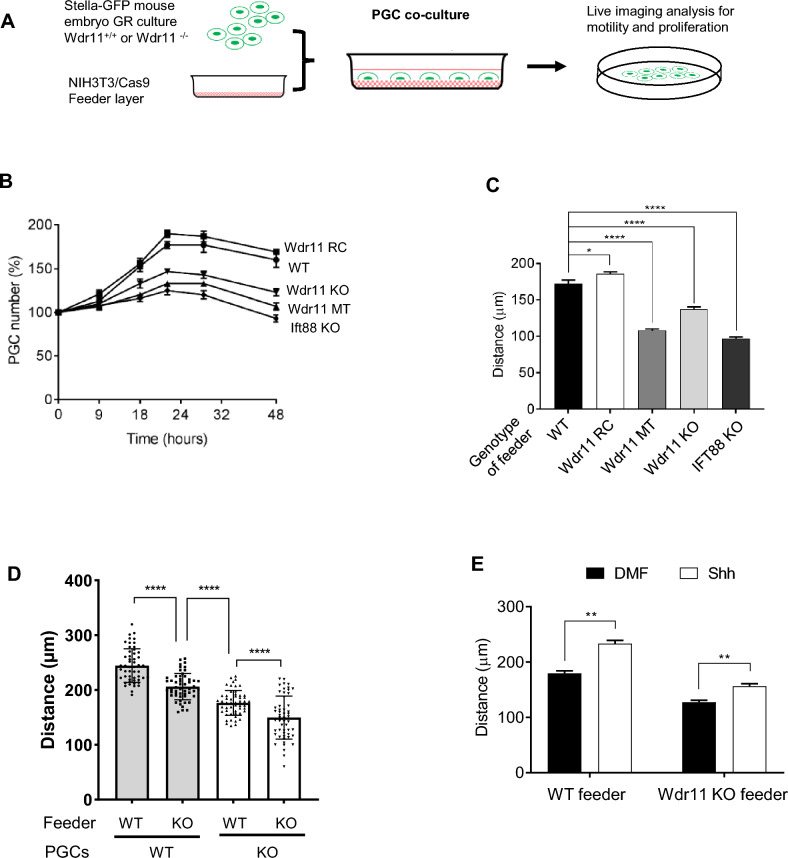
Effects of WDR11 mutations on PGC co-cultures. (**A**) Experimental scheme of PGC co-culture on NIH3T3/Cas9 feeders. (**B**) The proliferation of WT PGCs cultured on different NIH3T3/Cas9 feeders. The growth curves generated by counting GFP-positive cells from 10 random fields at the indicated time points. Percentage values shown as mean ± SEM were calculated from the total cell counts at 0 h. (**C**) Comparison of random motility of WT PGCs cultured on different NIH3T3/Cas9 feeders measured by time-lapse imaging (Supplementary Movies 3–7). The average accumulated moving distance of 20 GFP-positive cells in random fields tracked for 16 h in 3 biologically independent experiments is shown. Mean ± SEM after unpaired *t* test with Welch’s correction (*P < 0.01; ****P < 0.0001). (**D**) Comparison of random motility of WT or *Wdr11*^*–/–*^ PGCs cultured on WT or *Wdr11* KO NIH3T3/Cas9 feeders measured by time-lapse imaging. The average accumulated distance of 53 GFP-positive cells from each group was tracked for 16 h in random fields of view and analysed as above. (**E**) Effects of Shh-N on the motility of WT PGCs cultured on WT or *Wdr11* KO feeder measured by time-lapse imaging (Supplementary Movies 8–11) and analysed as above.

PGCs actively communicate with the neighbouring tissue for survival and expansion. The delayed migration and proliferation of *Wdr11*^*–*/–^ PGCs might be a net effect of three parallel events; (1) the defective ciliation of mesenchymal cells abolishing a canonical HH signal necessary for their own proliferation and support of PGCs; (2) reduced intrinsic motility and proliferation of PGCs through defective noncanonical HH signalling; (3) failed communication between PGCs and their somatic niche. We so far demonstrated the differential roles of WDR11 in PGCs and their niche, but whether WDR11 also modulates the communication between them is unclear. Intercellular communication via cilia-derived vesicle shedding has been reported^55^. So, we sought to determine if defective cilia in *Wdr11*^*–*/–^ could attenuate the interaction between PGCs and soma. To this end, we established a PGC co-culture system where single-cell suspensions of GR tissues were seeded on to NIH3T3/Cas9 feeder layers. Isolated mouse PGCs can be cultured on a fibroblast feeder cell layer treated with mitomycin C^56^. Such feeder cells are physiologically active but not proliferating, and can support PGC proliferation, motility and survival for at least 48–72 h (Fig. 8A). We first investigated whether the *Wdr11* genotype of feeder cells could influence the proliferation of WT PGCs. *Ift88* KO cells were also employed as a control for defective cilia. Growth curves generated by counting GFP-positive cells (PGCs) in the co-cultures over 48 h indicated that feeders with the *Wdr11*-MT mutation, *Wdr11* KO or *Ift*88 KO supported significantly less PGC proliferation than WT feeders. In contrast, *Wdr11*-RC feeders supported PGC proliferation almost as effectively as WT (Fig. 8B). These results indicate that *Wdr11* mutations can influence PGC growth by altering adjacent somatic niche. The fact that *Ift88* KO feeders showed a similar impairment in supporting PGCs suggests that the ciliation status of the surrounding somatic cells is indeed relevant.

### WDR11 mutations affect PGC motility in co-culture

To test for effects of feeder genotype on PGC motility, we analysed the random motility of individual PGCs by time-lapse imaging of co-cultures (Supplementary Movies 3–7). WT PGCs cultured on *Wdr11*-MT, *Wdr11* KO and *Ift*88 KO feeders had markedly reduced motility (accumulated distance over 10 h) compared to WT. Interestingly, PGCs on *Wdr11*-RC feeders showed slightly higher motility (Fig. 8C). Therefore, mutations of WDR11 may significantly impact PGC migration by altering their niche. We next compared the motility of *Wdr11*^*–*/–^ PGCs cultured on WT and *Wdr11* KO feeders, respectively. The data showed that the defective intrinsic motility of *Wdr11*^*–*/–^ PGCs was worsened by *Wdr11* KO feeders compared to WT feeders, and WT feeders could not fully rescue the defective motility of *Wdr11*^*–*/–^ PGCs (Fig. 8D). Thus, intrinsic defects of PGCs seemed to be as important as the environmental effects.

### SHH partially rescues PGC motility in co-culture

We previously showed that treatment with SHH or a SMO agonist could enhance PGC motility, while SMO antagonists such as cyclopamine and vismodegib inhibited it^11^. Hence, the reduced PGC motility in our co-culture system might be due to insufficient HH supply from the environment. To test this notion, we asked if additional ligand could improve WT PGC motility growing on WT or *Wdr11* KO feeders. Shh-N treatment increased PGC motility on the WT feeders by 30.2 ± 0.6%, while those on the *Wdr11* KO feeder were increased by 22.9 ± 0.7% (Fig. 8E). Therefore, Shh-N enhanced PGC motility regardless of feeders, and the defect of *Wdr11* KO feeders could be partially overcome by Shh-N.

## Discussion

Here we report a previously undescribed role for WDR11, a KS/CHH-associated gene, in development of the germ line with direct consequences in PGC development, advocating a new way of thinking and goals for the treatment of KS/CHH patients. Current doctrine is that CHH/KS is a hypothalamic and/or pituitary disease caused by inappropriate development or failed reactivation of gonadotrophin-releasing hormone (GnRH) neurons at puberty (a ‘secondary’ hypogonadism). Hence infertility in KS/CHH is routinely treated by gonadotropin replacement therapy^57^. Indeed, migration of GnRH neurones is disrupted in *Wdr11*^*–*/–^ mice causing reduced total numbers of GnRH neurones reaching the hypothalamus. However, current data suggest that defects within the gonads (a ‘primary’ hypogonadism) may exist in individuals with WDR11 mutations, especially those who failed to respond to gonadotropin therapy. It is noteworthy that genes known to regulate PGC migration such as the chemokine SDF1 and its receptor CXCR4 are also important in GnRH neuron migration, and decreased numbers of GnRH neurons are observed in Cxcr4 deficient mice^58^. Rare variants of GLI, SMO and PTCH1 have also been associated with KS/CHH^59–61^, so the migration of developing GnRH neurones and PGCs are linked and may be mediated by overlapping signalling pathways.

How noncanonical HH signalling pathway is determined or spatiotemporally regulated remained unclear. We previously demonstrated that GAS1 receives and presents SHH to PTCH2, inducing rapid induction of p-CREB and p-Src. Loss of GAS1 abolished PTCH2-mediated noncanonical signalling but not PTCH1-mediated responses^11^. Here we report the first evidence that GAS1 itself is a specific target of PTCH2-mediated HH signalling, further defining the noncanonical pathway. Moreover, GAS1, but not BOC and CDO, can be released by shedding in PTCH1-expressing NIH3T3 cells. Intriguingly, ADAM17 involved in the release of membrane-tethered SHH essential for the establishment of morphogen gradient^54^ may also regulate GAS1 shedding (Fig. 6). Further research is required to confirm if GAS1 is indeed a substrate for ADAM17 and if so, whether they operate differentially in different receptor contexts. It is tempting to speculate that GAS1 and SHH may be solubilised by a common mechanism, providing another link for noncanonical and canonical pathway.

We also show that ciliary translocation of SMO occurs only in PTCH1/BOC(CDO) context, while PTCH2/GAS1 associates with SMO outside cilia (Fig. 7). Such compartmentalisation may mediate differential SMO signalling through access to different binding partners and effectors. We also propose that BOC/CDO may serve as the ciliary anchor for PTCH1. SHH binding elicits PTCH1 dissociation from BOC/CDO which constitutively reside in the cilia. After dissociation, PTCH1 is expelled from cilia, allowing SMO entry, a critical step for canonical HH signalling. Although PTCH1 is believed to possess a putative ciliary localisation signal, deletion of this region did not abolish SMO repression^62^, indicating the existence of an additional mechanism. Our study provides novel insight into the differential functions of BOC/CDO and GAS1, the obligatory coreceptors of HH. Further investigation of the molecular domains involved and their effects on the signalling outcome anticipates.

Unciliated PGCs likely stay insensitive to the developmental programs driven by canonical HH signalling. Our study shows WDR11 regulates both the PGC-autonomous (cilia-independent) and PGC-nonautonomous (cilia-dependent) events. Loss of WDR11 disrupted PGC migration/proliferation driven by PTCH2-dependent noncanonical HH signalling, but the exact function of WDR11 in noncanonical HH pathway remains unclear. An involvement in the trafficking of receptors outside of cilia seems a likely scenario. WDR11 may contribute to the PTCH1-dependent canonical HH signalling via at least two mechanisms. First, we previously demonstrate that WDR11 binds both full-length (FL) and cleaved repressor (R) forms of GLI3. *Wdr11*^*–*/–^ cells accumulate GLI3FL incapable of stimulating downstream signals, indicating a defective GLI processing, but overexpression of WDR11 alone without HH stimulation did not induce GLI activation^35^. WDR11 locates broadly in the cytoplasm, both as a perinuclear cloud of dots consistent with a TGN localisation and as a peripheral punctate pattern suggestive of endosomes^28,35,63,64^. SMO agonist enhanced WDR11 localisation to the basal body, while SMO antagonist arrested it at the perinuclear location^35^. Therefore, WDR11 may facilitate the processing and trafficking of GLI to and from the cilia in response to HH signalling. Intriguingly, WDR11 has been linked with the networks of lysosomes and autophagosomes^29,30^. Second, WDR11 may facilitate canonical HH signalling by promoting ciliogenesis. WDR11 localises at the base of cilia where the IFT particles are assembled before moving into the cilium^35,65^. WDR11 may shuttle between the TGN and the basal body to mediate trafficking of ciliary proteins such as IFT20, essential for axoneme extension. Notably, IFT is also involved in the processing/function of GLI^66^. Independent studies have shown that SMO-dependent noncanonical HH signalling can induce ciliogenesis^15,67^. Since WDR11 expression is induced by SMO-dependent noncanonical HH signalling^35^, WDR11 may be one of such cooperative links between the noncanonical and canonical pathways during ciliogenesis.

WDR11 may regulate IFT-B trafficking as evidenced by the altered localisation profile of IFT88 and IFT57 in WDR11 KO cells (Fig. 3). The IFT-B complex consists of different sub-complexes. IFT88 is part of the core IFT-B, while IFT20 and IFT57 are part of the peripheral IFT-B which serves as a linker between the kinesin motor and the core IFT-B^19^. Although WDR11 was co-precipitated with IFT57 and IFT88, we could not conclude whether WDR11 binds directly with these proteins independently of IFT20, and whether all IFT proteins are expressed at normal levels in WDR11 KO cells. It is likely that WDR11 plays a role in the targeted recruitment of IFT20 and its interacting proteins from the TGN to the basal body, indirectly modulating the function of the IFT-B sub-complexes. GMAP210, a member of the golgins, was proposed to be responsible for the recruitment of cytosolic IFT20 to the Golgi^44^. However, a molecular interaction study using BioID did not identify GMAP210 as an interactant of IFT20^68^. Further study is necessary to determine the exact nature of these interactions and if WDR11 and IFT20 indeed share a specific endosome vesicle trafficking pathway.

## Materials and methods

### Breeding of transgenic mice

*Stella*^*GFP*^ mice were originally obtained from Azim Surani (Gurdon Institute, Cambridge, UK) and maintained in a C57BL/6 background as described^11^. The *Wdr11* KO mouse (International Gene Trap Consortium Ayu21-KBW205) was generated at the Institute of Resource Development and Analysis, Kumamoto University in Japan^35^. To establish the *Stella*^*GFP*+*/*+^*;Wdr11*^+*/–*^ hybrid line, homozygous *Stella*^*GFP*^ mice were crossed with *Wdr11*^+*/–*^ mice. The noon copulation plug was counted as embryonic day 0.5 after timed mating. All experiments were conducted in accordance with the Animals (Scientific Procedures) Act 1986 in the Biological Research Facility at St. George’s, University of London (PPL 70/8512) according to the rules and protocols approved by St. George’s, University of London, Research Ethics Committee and the Genetic Modification Committee, complied with the ARRIVE guidelines.

### Mouse genotyping

Genotypes were verified by PCR and qPCR analyses of the genomic DNA. The copy number of the GFP allele was determined by qPCR to confirm genotypes. To perform relative quantification, the crossing point (Cp) value of the target gene (*GFP*) was normalised to the Cp of the reference gene (*β-tubulin*), based on which the Relative Copy Number Ratios (RCNR) were generated. Copy Number Variation (CNV) was calculated by $CNV=\frac{1}{baseline \,\,RCNR}\times Targeted \,\,gene \,\,RCNR$*.* A rounded CNV value of 1 was considered to indicate a heterozygote, 2 a homozygote and 0 a WT mouse (no GFP). The presence of the *Stella*^*GFP*^ allele was further confirmed by test-breeding of randomly selected homozygous litters with WT mice, followed by PCR amplification of GFP. Primers used for genotyping analyses are shown in Supplementary Information.

### qPCR and RT-PCR

For RT-PCR analyses, mouse tissues were harvested and homogenized before total RNA was extracted using a RNeasy Mini Kit according to the manufacturer’s protocol. First-strand complementary DNA (cDNA) was synthesized using oligo(dT) primers and the Precision nanoScript2 Reverse Transcription Kit (Primer Design). Quantitative real-time PCR was performed using the Maxima® SYBR green qPCR master mix (Thermo Fisher Scientific) in a Light Cycler 2.0 instrument (Roche). The crossing point (Cp) values were obtained by LightCycler® Version 4.1 software (Roche). Cp values were analysed using the 2^-ΔΔCT^ method normalised to *Gapdh*. All primers used are provided in Supplementary Information.

### Immunohistochemistry

Embryos were fixed in 4% paraformaldehyde before paraffin embedding. Sections cut at 6 μm thickness were deparaffinised with Histoclear (National Diagnostics) and rehydrated in PBS. For β-galactosidase detection, whole-mount embryos were fixed with X-gal Fix buffer (0.2% glutaraldehyde, 2% paraformaldehyde, 5 mM EGTA, 2 mM MgCl_2_ in PBS pH 7.4) for 1 h at 4 °C, washed in PBS and then incubated overnight at 37 °C in X-gal solution (1 mg/ml X-gal, 2 mM MgCl_2_, 5 mM K3Fe(CN)6 in PBS at pH 7.4). After washing in PBS and paraffin-embedding, samples were sectioned at 12 µm-thickness and counterstained with eosin. For alkaline phosphatase (ALP) staining, embryo sections were stained with BCIP-NBT (Roche) in ALP buffer at 4 °C. Images of embryo sections were analysed by Zeiss Axioplan 2 Upright.

### Immunofluorescence staining of GR sections

Serial sections of dissected embryos at 5–7 μm thickness were deparaffinized, rehydrated and washed in PBS. Following antigen retrieval in sodium citrate buffer (10 mM sodium citrate, 0.05% Tween 20, pH 6.0), sections were blocked with 10% goat serum in 0.5% Triton-X PBS for 1 h at room temperature and then incubated overnight at 4 °C with primary antibodies diluted in 10% goat serum in 0.5% Tween in PBS. After washing, samples were incubated with fluorescence-labelled secondary antibodies at 1:500 dilution and counterstained with DAPI before mounting in Mowiol. For immunofluorescence analyses of cultured cells, cells were plated on glass coverslips, fixed with 4% PFA, permeabilized with 0.2% Triton X‑100 in PBS, and incubated in blocking buffer (2% heat-inactivated goat serum, 0.2% Triton X‑100 in PBS) before probing with primary antibodies diluted in blocking buffer. After washing, secondary antibodies were added along with DAPI. Fluorescence microscopy was performed using a Zeiss Axiovert 200 M Upright microscope and analysed by ImageJ software (http://rsbweb.nih.gov/ij/). The total number of PGCs was generated by counting SSEA-positive cells from every other slide of the serial sections of E9.5, E10.5 and E11.5 embryos.

### Imaging and analyses of primary cilia and F-actin

Cultured cells on glass cover slips were serum-starved for 18–24 h before fixing to induce ciliogenesis. Samples were analysed by immunofluorescence staining with an anti-ARL13B antibody that visualises the cilia axoneme or an anti-gamma-tubulin antibody that visualises the basal body, along with DAPI staining for nucleus. To generate ciliation frequency values, the total number of cilia and nuclei were counted from the maximum intensity projection images of each channel manually. The length of cilia was assessed in random fields of cells by measuring the maximum projection using ImageJ. Whole sections of embryos prepared as above were stained with antibodies against ARL13B and SSEA1 and the cilia were analysed from every 4th slides. To generate the 3D imaging of the GR section, 3D volume rendering of the image stacks was performed in ImageJ software using the volume viewer plugin. For F-actin staining, Alexa Fluor 488-conjugated phalloidin (Invitrogen, LSA12379) was used.

### Embryo slice culture and live imaging

Embryo slice organ culture and filming were performed as previously described^11^. Briefly, transverse sections of E10.5 embryos were cultured in Hepes-buffered DMEM/F-12 medium with 0.04% lipid-free BSA and 100U/ml penicillin/streptomycin. A single optical section was captured every 15 min for approximately 10 h (a total of 40 frames). The z-stack images were extracted as TIFF files and one stack per time interval was put together using ImageJ to create a movie. Motile behaviour of PGCs was evaluated based on accumulated distance (total cell path travelled), Euclidean distance (the shortest distance between cell start and end points), cell velocity and directionality (the ratio between Euclidean distance and accumulated distance indicating the straightness of the migration path) using the Chemotaxis and Migration Tool 2.0 plug-in software (Ibidi GmbH). Velocity measurements were generated for each time interval by using the formula V = [sqrt (dx^2^ + dy^2^)](p)/0.25 h, where dx is the change in the x-axis, dy is the change in the y-axis, and p is the pixel size in µm. The velocities of all the tracked cells were averaged to obtain an overall mean velocity for each embryo slice/movie. Tracking was performed only on those PGCs that remained in focus and viable for the entire duration of filming. Ectopic PGCs localised in the mesentery and hindgut were not analysed as they tend to disintegrate during filming. Cell survival was assessed by the number of hours that the GFP fluorescence from a cell was detected during the imaging. We confirmed the developmental stages of the embryos used in our analyses by anatomical morphology landmarks such as somite numbers, absence/presence of hind limbs and tail buds (E9.5 and E10.5, respectively), the complete closure of lens vesicle (E11.5) and finger rays and retinal pigmentation (E12.5), which indicated that mutant embryos did not have general developmental defects affecting the migration of PGCs, at least during the period we studied. *Wdr11* KO embryos show lethality only after E12.5 as we previously reported, therefore there shouldn’t have been any significant defects during E9.5–11.5 which was our main focus.

### Genital ridge primary culture and live imaging

Dissected GR tissues of E10.5 embryos were digested in 0.25% trypsin, passed through a 0.4 μm cell strainer and suspended in DMEM/L-15 medium supplemented with 20% knockout serum replacement (Invitrogen), 2 mM l-glutamine, 0.1 mM non-essential amino acids and 0.1 mM 2-mercaptoethanol (Sigma-Aldrich), before being plated onto 0.1% gelatin-coated cover slips. Cells were incubated in 0.5% serum-containing media before treatment with 200 ng/mL recombinant SHH N-terminal peptide (R&D Systems, 1314-SH) diluted in dimethyl formamide (DMF).

For PGC co-cultures with feeder layers, single-cell suspensions generated from dissected GR tissues were plated onto the NIH3T3/Cas9 feeder layer pre-treated with Mitomycin-C (5 μg/ml). The proliferation and motility of PGCs were measured by time-lapse imaging of GFP-positive cells captured every 15 min for 10 h. Live imaging was performed using Nikon A1R laser scanning confocal microscope in a humidified 5% CO_2_ chamber at 37.0 ± 0.5 °C. Random motility of PGC was analyzed using the Chemotaxis and Migration Tool 2.0 plug-in software (Ibidi GmbH).

### NIH3T3 cell culture, CRISPR/Cas9 and plasmid constructs

NIH 3T3 cells (American Type Culture Collection, Manassas, VA) were routinely cultured in DMEM with 2 mM l-glutamine and 100 µg/ml penicillin/streptomycin (Sigma-Aldrich), supplemented with 10% newborn calf serum (NCS). For growth curve analyses, NIH3T3 cells were plated at 2 × 10^6^ cells per 10 cm dish in the growth medium and total cell counts were assessed every 12 h. NIH3T3 cells with targeted editing of *Wdr11* and *Ift*88 were generated using the CRISPR/Cas9 approach. Briefly, sgRNAs designed using the CRISPR Design Tool (http://crispr.mit.edu) were cloned into pSpCas9(BB)-2A-Puro (Addgene #48139) and transfected using Polyfect (Promega). To isolate single-cell clones, transfected cells were plated in 96-well plates. After selection in puromycin (Cambridge Bioscience), positive clones were confirmed by Sanger sequencing and western blot. The sequences of the sgRNA and primers used are provided in Supplementary Information. NIH3T3/Cas9 cells with targeted deletion of PTCH1 (endogenous PTCH2 is absent) as previously described^11^ were transfected with pcDNA3-mPtch1 and pCMV6-mPtch2-MycDDK (Origene). For overexpression in WT NIH3T3 cells, the full-length WDR11 expression construct made in pcDEST-Myc as previously described^35^ was transfected. The full-length human SHH expression construct (pCS2-Shh) was a kind gift from Adrian Salic. For cilia analyses, NIH3T3/Cas9 cells plated onto the glass cover slips coated with 0.001% poly-L-lysine in PBS were incubated in a serum-free medium for 24 h to induce primary cilia formation and the cilia length was measured after Arl13b immunostaining using ImageJ.

### Apoptosis and proliferation analyses

SSEA1-positive PGCs with co-localised staining of phosphohistone-H3 and cleaved caspase-3 were counted from every other section of the entire length of the gonadal ridge of E10.5 embryos from each genotype. The percentage values are obtained by manually counting the total positive cells against the total cell counts labelled with DAPI. DAPI-positive cells negative for SSEA1 were counted as somatic cells. The PGC growth curves were generated by counting GFP-positive cells from 10 random fields of GR primary cultures plated on the NIH3T3 feeder layer at 0, 9, 18, 24, 32 and 48 h after plating. The percentage fold was calculated from the total cell count at 0 h. The images were captured using an Olympus IX70 inverted microscope (Hamamatsu C4742–95, Hamamatsu, Japan).

### Western blot and co-immunoprecipitation

Total protein extracted in a lysis buffer (50 mM HEPES, 150 mM NaCl, 10% glycerol, 1% Nonidet P-40, and 1 mM EDTA) containing protease/phosphatase inhibitors (Sigma-Aldrich) was separated by SDS-PAGE and transferred onto Hybond-ECL membrane (Amersham) before being probed with primary antibodies diluted in blocking buffer (5% skim milk in TBS with 0.05% Tween 20 (TBST)). After washing in TBST, the membrane was incubated with horseradish peroxidase-conjugated secondary antibodies before analyses by enhanced chemiluminescence (GE Healthcare). Co-immunoprecipitation was performed using pre-cleared lysate (1–2 mg protein) and the immune complexes captured on protein A/G-Agarose beads (Santa Cruz Biotechnology) were analysed by Western blot. Conditioned medium was collected from NIH3T3 cultured in 0.5% serum-containing medium for 48 h with or without TMI005 (Sigma-Aldrich) and concentrated using Vivaspin 6 with 10 kDa MWCO (Sigma-Aldrich).

### Antibodies

Primary antibodies used were against GFP (Rabbit IgG, 1:200, Abcam, ab290), SSEA1 (Mouse IgG, 1:200, Developmental Studies Hybridoma Bank, MC-480), Stella (Rabbit IgG, 1:200, Abcam, ab19878), phospho-histone H3 (Rabbit IgG, 1:500, Millipore, 06-570), cleaved-caspase 3 (Rabbit IgG, 1:200, Cell Signalling, 9661), ARL13B (Rabbit IgG, 1:1000, Proteintech, 17711‐1‐AP), gamma‑tubulin (mouse IgG, 1:1000, Sigma T6557), acetylated alpha‑tubulin (Sigma T6793), phospho-Src (Rabbit IgG, 1:200, Invitrogen, 44-660G), phospho-CREB (Rabbit IgG, 1:200, Cell Signaling, 9198), WDR11 (rabbit IgG, 1:100, Abcam, ab175256; goat IgG, 1:100, Santa Cruz, sc-163523), IFT88 (rabbit IgG, 1:500, Proteintech, 13967-1-AP), IFT140 (rabbit IgG, 1:500, Proteintech, 17460-1-AP), IFT57 (rabbit IgG, 1:500, Proteintech, 11083-1-AP), IFT20 (rabbit IgG, 1:500, Proteintech, 13615-1-AP), HHIP1 (mouse monoclonal, 1:1000, Santa Cruz, sc-47754), MYC (M4439, mouse monoclonal 1:200, Sigma-Aldrich), PTCH1 (sc-293416, mouse monoclonal 1:200, Santa Cruz), PTCH2 (PA1-46223, rabbit polyclonal 1:200, Invitrogen), GAS1 (AF2636, goat polyclonal 1:500, R&D; PA5-48298, rabbit polyclonal 1:500), BOC (AF2385, goat polyclonal 1:500, R&D; MAB20361, mouse monoclonal 1:500, R&D), CDO (GTX105422, rabbit polyclonal 1:500, GeneTex), SMO (sc-166685, mouse monoclonal 1:200, Santa Cruz), CEP164 (sc-515403, mouse monoclonal 1:200, Santa cruz), ADAM17 (sc-390859, mouse monoclonal 1:200, Santa cruz) and b-actin (rabbit IgG, 1:500, CST, 4967L). Secondary antibodies, all of which were from Invitrogen Thermo Fisher Scientific and used at 1:5000 dilution, include Alexa Fluor 488 (Goat anti‐rabbit, A-11008; Donkey anti-goat, A-11055), Alexa Fluor 555 (Goat anti‐mouse, A-21422; Goat anti-rabbit, A-27039), and Alexa Fluor 568 (Goat anti‐rabbit, A-11011).

### Statistical analyses

Statistical analyses were performed using GraphPad Prism 5 (La Jolla, CA, USA). The numbers of independently replicated experiments (n) are indicated in the relevant figure legends where possible. In some experiments where percentage values are indicated, the values were calculated from the average raw data value of the sample divided by the average raw data value of the control. Significance was tested using an unpaired student’s *t* test. In experiments where the number of repeated measures was unequal per group, a one-way analysis of variance (ANOVA) was used with Welch’s test.

## Supplementary Information

Supplementary Figures.

Supplementary Information 1.

Supplementary Figures.

Supplementary Information 2.

Supplementary Video 1.

Supplementary Video 2.

Supplementary Video 3.

Supplementary Video 4.

Supplementary Video 5.

Supplementary Video 6.

Supplementary Video 7.

Supplementary Video 8.

Supplementary Video 9.

Supplementary Video 10.

Supplementary Video 11.

## Data Availability

All data generated or analysed during this study are included in this published article and its supplementary information files.
