# Supplementary material for: Coordination of canonical and noncanonical Hedgehog signalling pathways mediated by WDR11 during primordial germ cell development
Source: Sci Rep. 2023 Jul 29;13:12309. doi: 10.1038/s41598-023-38017-9 (PMC10387110; doi:10.1038/s41598-023-38017-9)
Supplement: Supplementary file 1 — Supplementary Figures. [file 41598_2023_38017_MOESM1_ESM.pptx]

## Slide 1
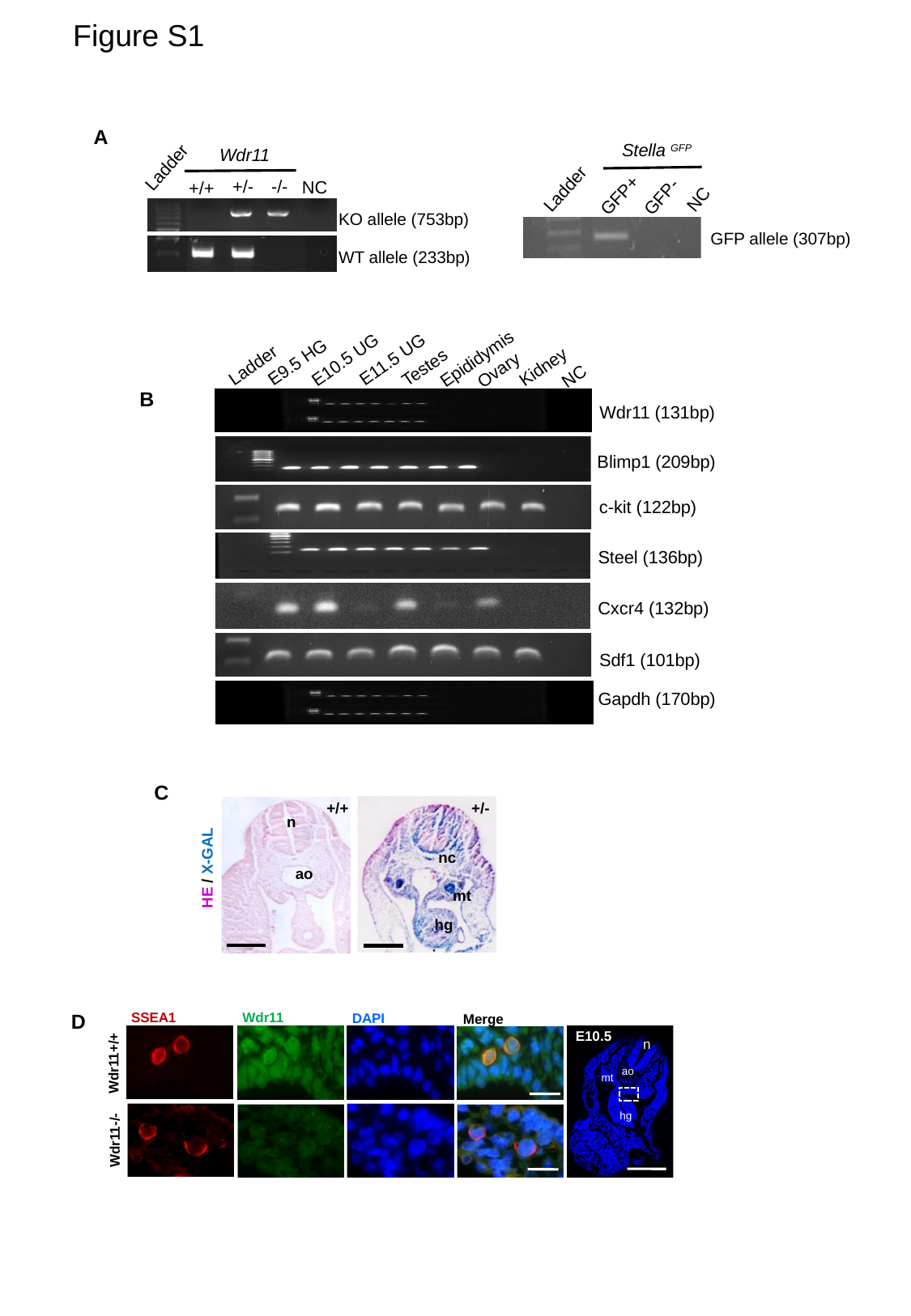

Figure S1
A
Wdr11
Ladder
-/-
+/-
NC
 +/+
KO allele (753bp)
WT allele (233bp)
Stella GFP
Ladder
GFP+
GFP-
NC
 GFP allele (307bp)
Epididymis
E10.5 UG
E9.5 HG
Kidney
Testes
Ovary
NC
B
Wdr11 (131bp)
Blimp1 (209bp)
c-kit (122bp)
Steel (136bp)
Cxcr4 (132bp)
Sdf1 (101bp)
Gapdh (170bp)
E11.5 UG
Ladder
C
+/-
+/+
n
nc
ao
mt
hg
HE / X-GAL
Wdr11
SSEA1
DAPI
Merge
E10.5
Wdr11+/+
Wdr11-/-
D
n
ao
mt
hg

## Slide 2
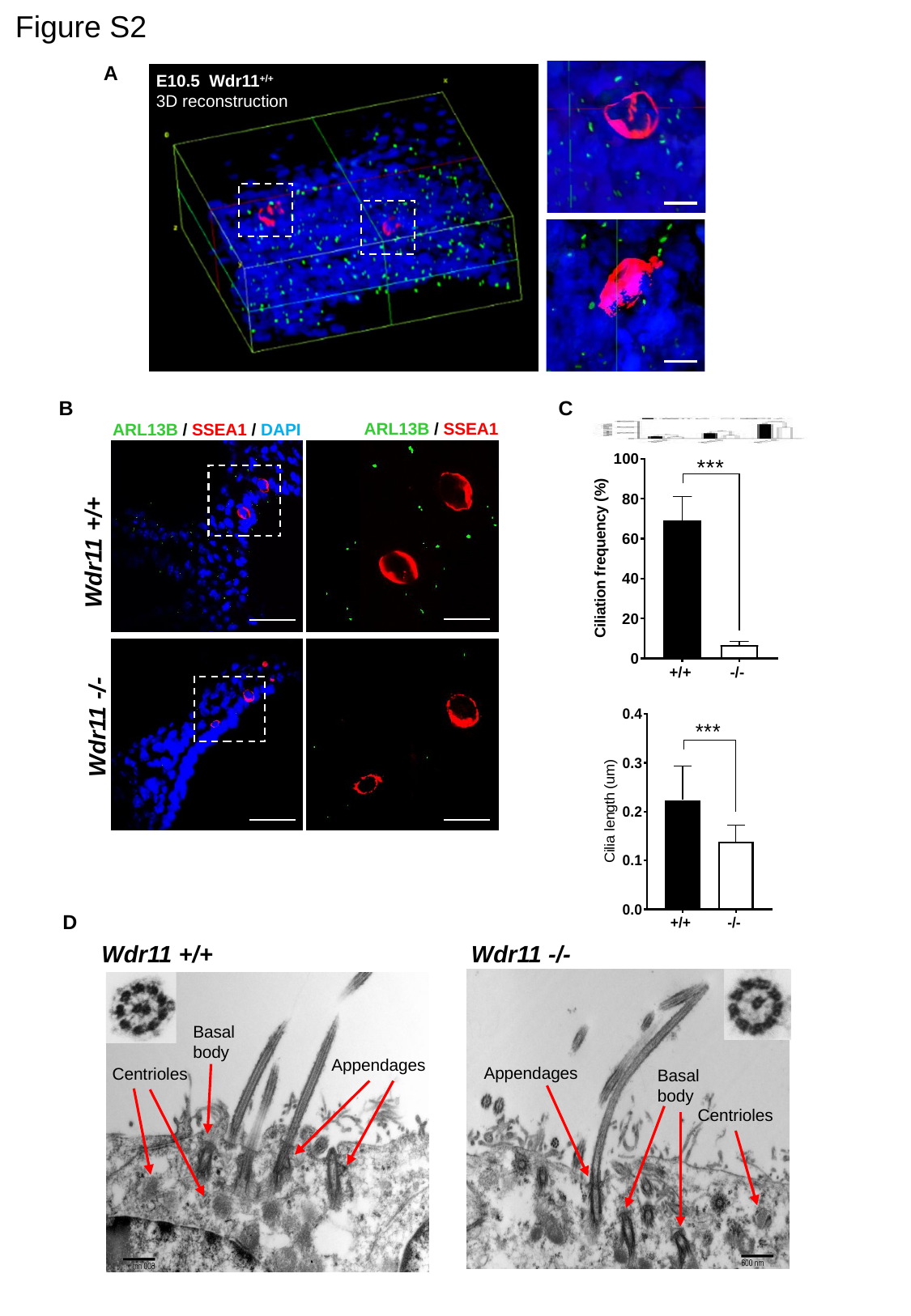

Figure S2
A
E10.5 Wdr11+/+
3D reconstruction
B
C
ARL13B / SSEA1
ARL13B / SSEA1 / DAPI
Wdr11 -/-
Wdr11 +/+
D
Wdr11 +/+
Wdr11 -/-
Appendages
Basal
body
Centrioles
Basal
body
Appendages
Centrioles

## Slide 3
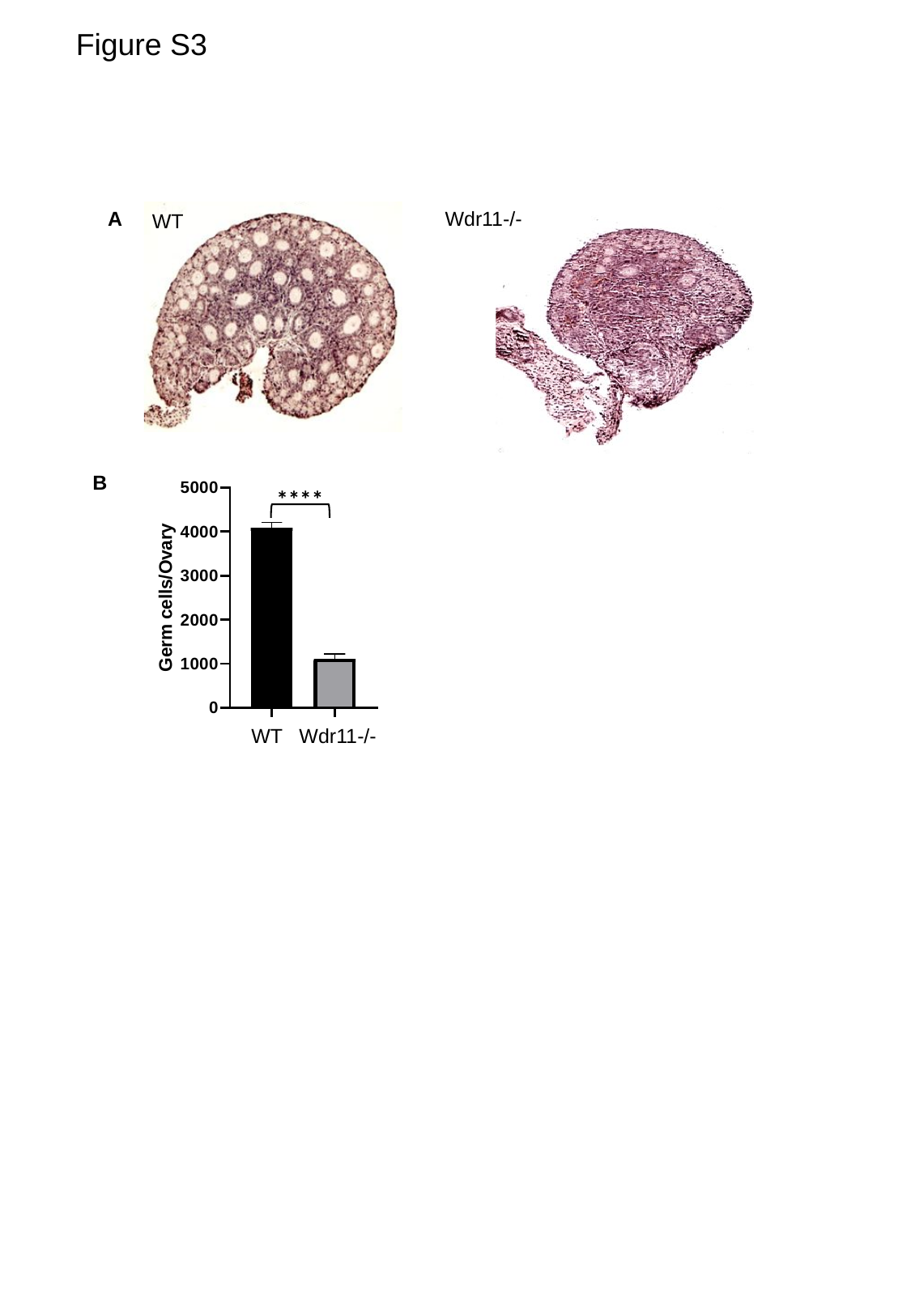

Figure S3
Wdr11-/-
A
WT
****
WT Wdr11-/-
WT
Wdr11-/-
B

## Slide 4
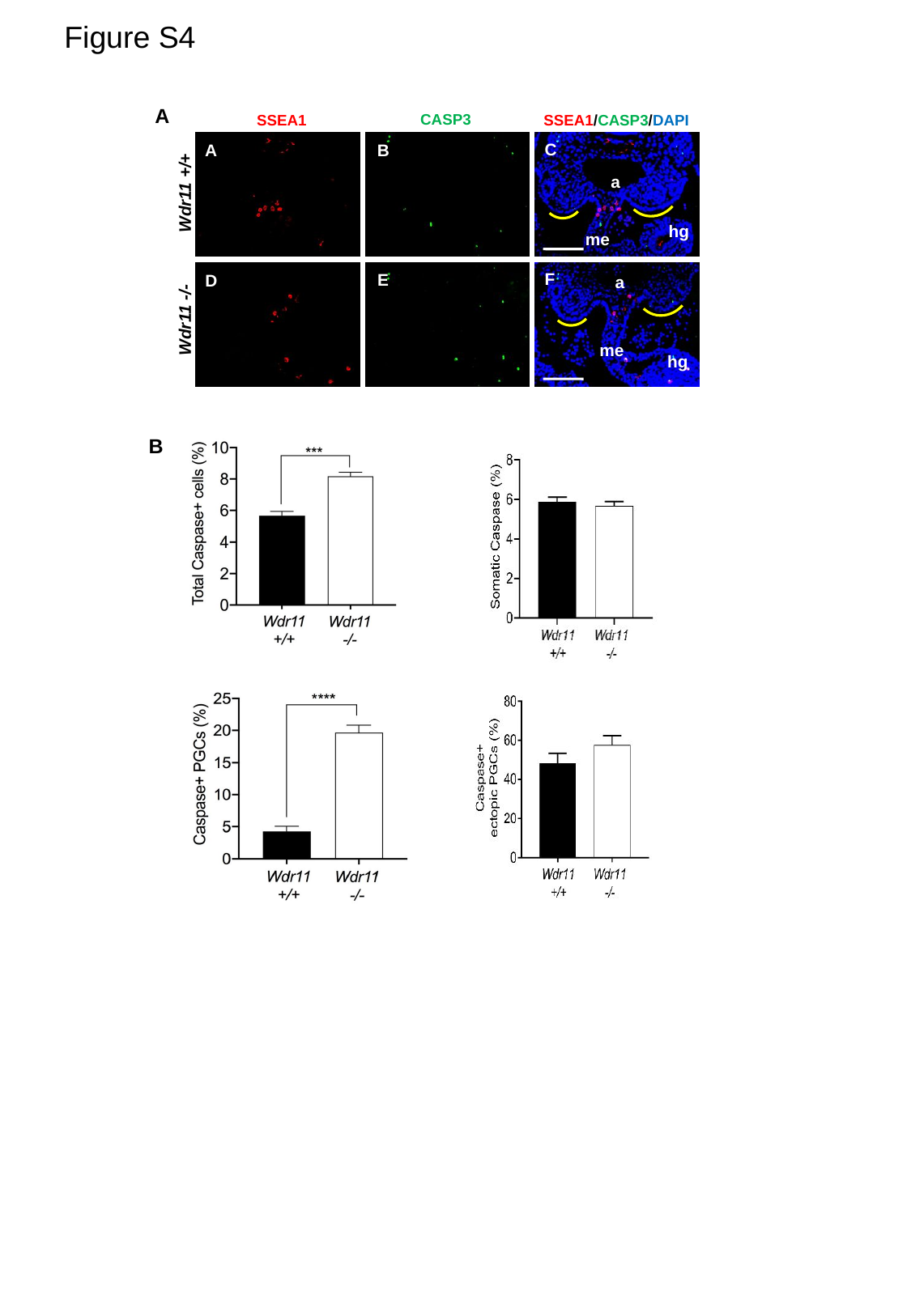

Figure S4
A
CASP3
SSEA1/CASP3/DAPI
SSEA1
C
B
A
a
Wdr11 +/+
hg
me
F
E
D
a
Wdr11 -/-
me
hg
B

## Slide 5
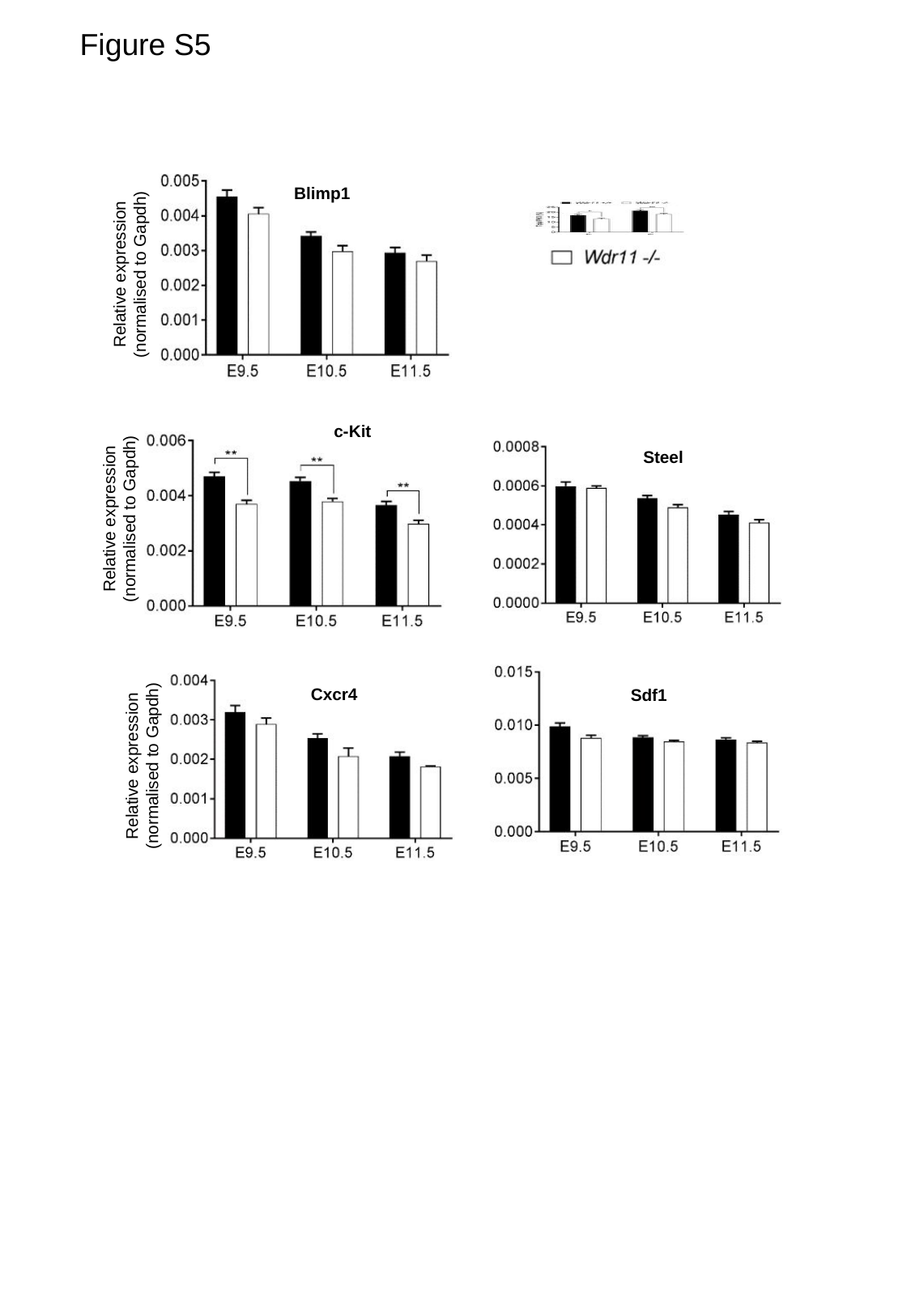

Figure S5
Relative expression
(normalised to Gapdh)
Blimp1
c-Kit
Steel
Relative expression
(normalised to Gapdh)
Sdf1
Cxcr4
Relative expression
(normalised to Gapdh)

## Slide 6
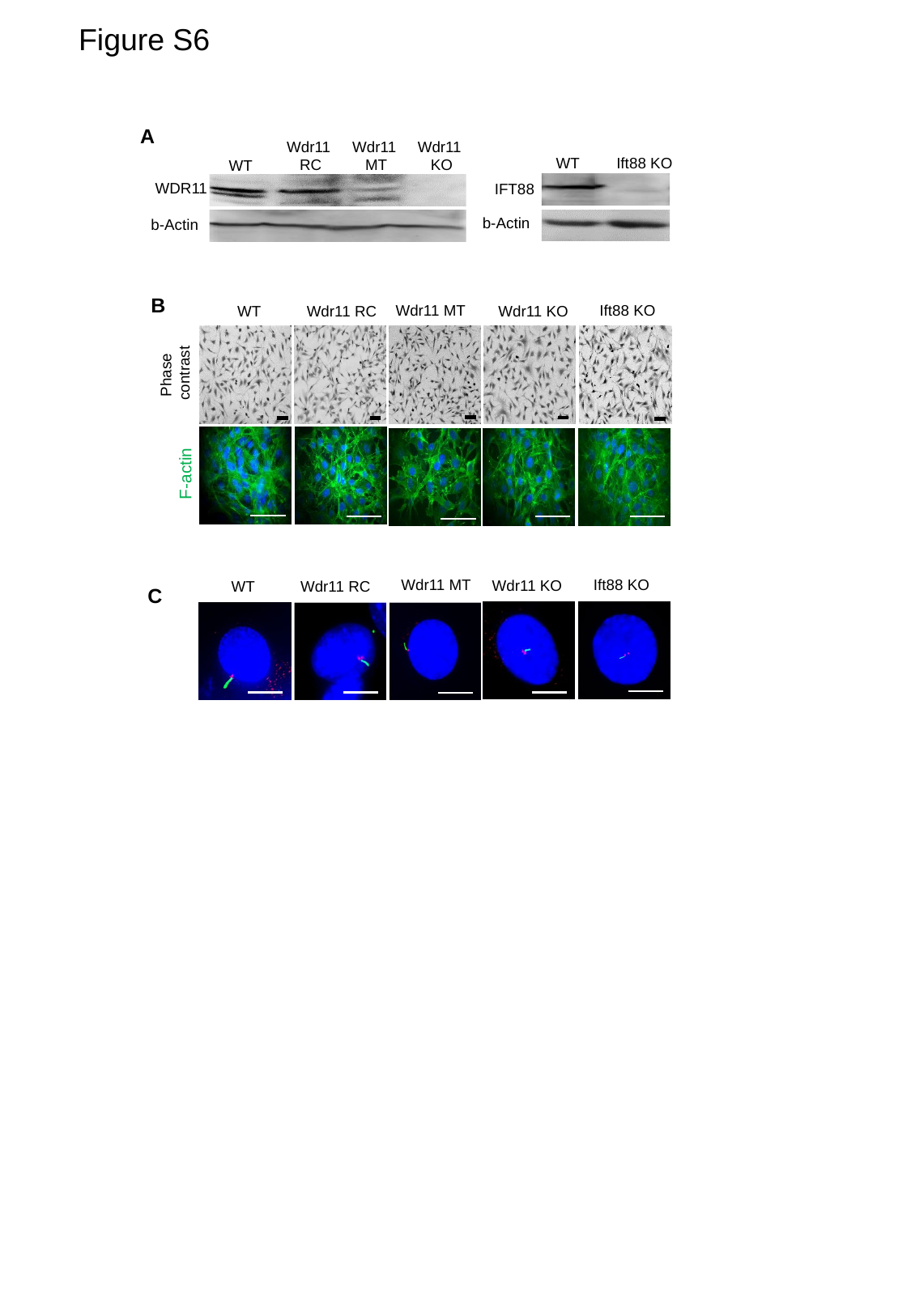

Figure S6
A
Wdr11
RC
Wdr11
MT
WT
Ift88 KO
WT
WDR11
IFT88
b-Actin
b-Actin
Wdr11
KO
B
Wdr11 MT
Ift88 KO
Wdr11 KO
WT
Wdr11 RC
Phase
 contrast
F-actin
Wdr11 MT
Ift88 KO
Wdr11 KO
WT
Wdr11 RC
C

## Slide 7
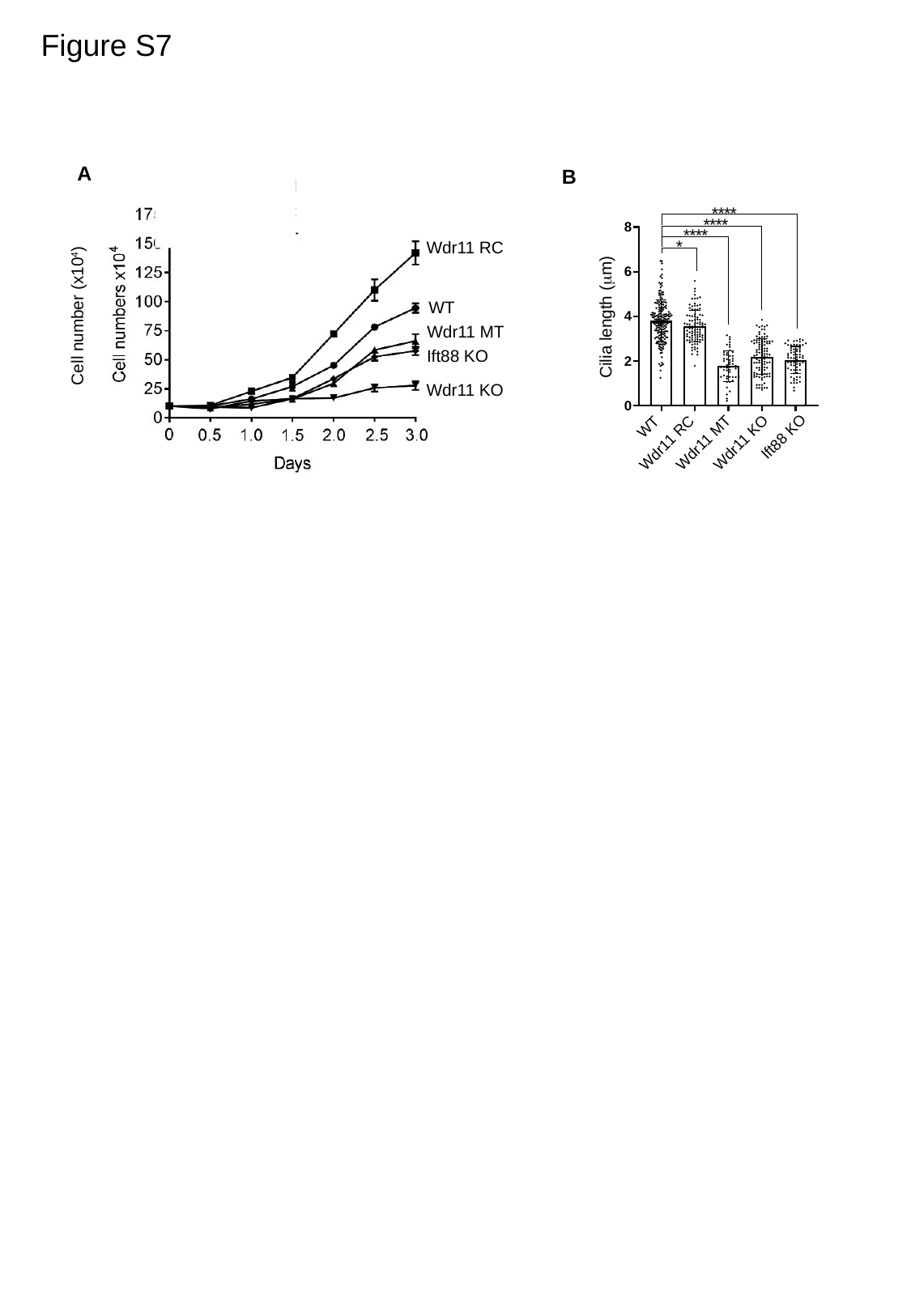

Figure S7
A
Wdr11 RC
WT
Wdr11 MT
Ift88 KO
Wdr11 KO
Cell number (x104)
B
Cilia length (mm)
WT
Ift88 KO
Wdr11 KO
Wdr11 MT
Wdr11 RC

## Slide 8
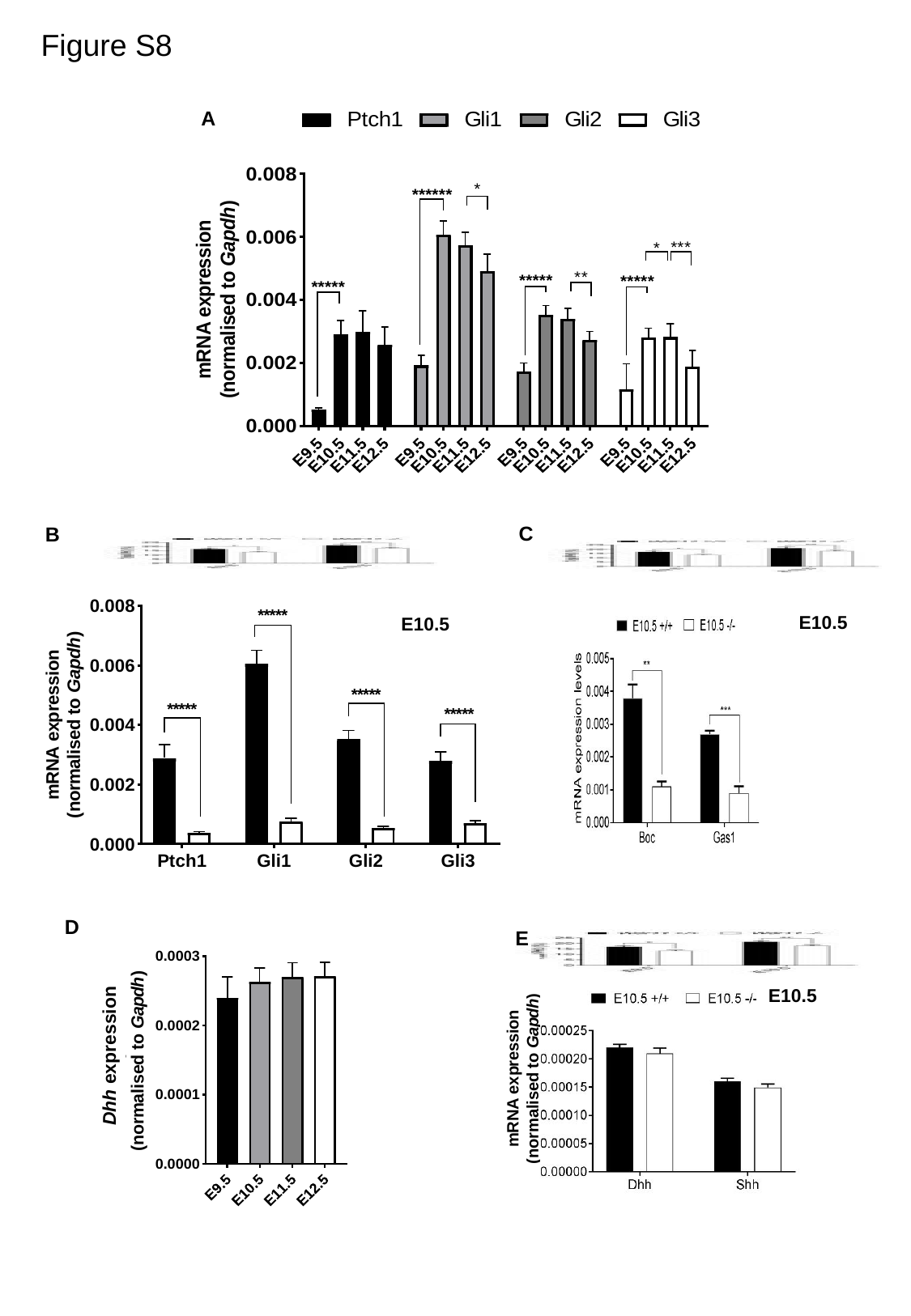

Figure S8
A
C
E10.5
B
E10.5
D
 Dhh expression
E
E10.5

## Slide 9
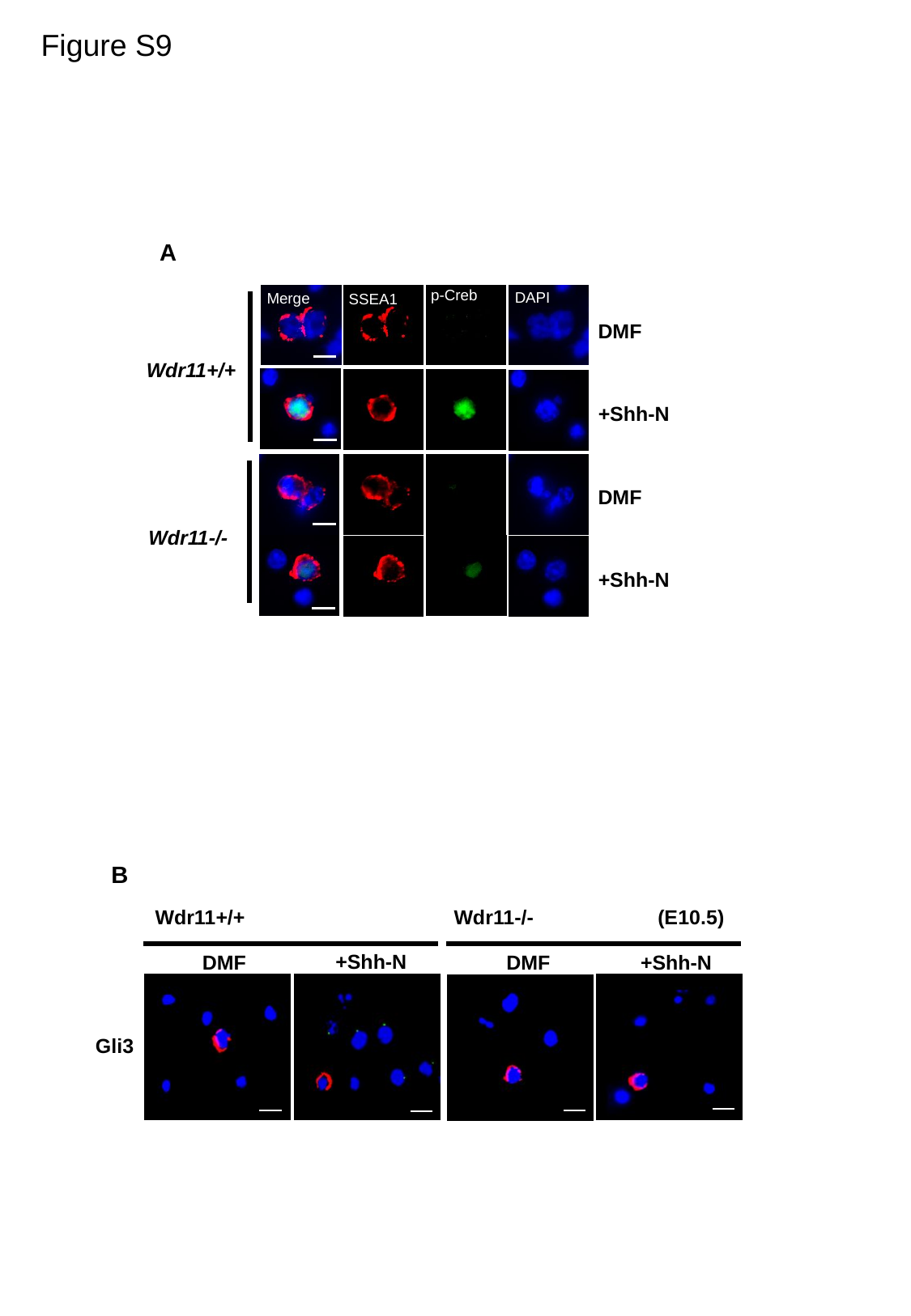

Figure S9
A
p-Creb
DAPI
Merge
SSEA1
DMF
Wdr11+/+
+Shh-N
DMF
Wdr11-/-
+Shh-N
B
Wdr11+/+ Wdr11-/- (E10.5)
+Shh-N
DMF
DMF
+Shh-N
Gli3
