# Supplementary material for: Coordination of canonical and noncanonical Hedgehog signalling pathways mediated by WDR11 during primordial germ cell development
Source: Sci Rep. 2023 Jul 29;13:12309. doi: 10.1038/s41598-023-38017-9 (PMC10387110; doi:10.1038/s41598-023-38017-9)
Supplement: Supplementary file 2 — Supplementary Information 1. [file 41598_2023_38017_MOESM2_ESM.docx]

**Coordination of canonical and noncanonical Hedgehog signalling pathways mediated by WDR11 during primordial germ cell development**

Jiyoung Lee^1, ‡, ∞^, Yeonjoo Kim^1, #, ∞^, Paris Ataliotis^1^, Hyung-Goo Kim^2^, Dae-Won Kim^3^, Dorothy C. Bennett^1^, Nigel A. Brown^1^, Lawrence C. Layman^4^ and Soo-Hyun Kim^1^*

^1^Molecular and Clinical Sciences Research Institute, St. George’s, University of London, London, UK; ^2^Neurological Disorders Research Center, Qatar Biomedical Research Institute, Hamad Bin Khalifa University, Doha, Qatar; ^3^Department of Biochemistry, Yonsei University, Seoul, Republic of Korea; ^4^Section of Reproductive Endocrinology, Infertility and Genetics; Department of Obstetrics and Gynecology; Department of Neuroscience and Regenerative Medicine; Department of Physiology, Medical College of Georgia, Augusta University, Augusta, USA.

^‡^Current affiliation: Department of Haematology, Wellcome-MRC Cambridge Stem Cell Institute, University of Cambridge, UK

^#^Current affiliation: The Babraham Institute, Cambridge, UK

∞ These authors contributed equally.

**SUPPLEMENTARY TABLES**

**Supplementary Table 1. List of primers used for mouse genotyping**

| **Gene** | **Genebank** | **Tm (°C)** | **Primer sequences (5’ to 3’)** | **Product size** |
| --- | --- | --- | --- | --- |
| WT allele | NM_172255.3 | 58 | F: ATGGCCTGGGATTTGATGACC  R: AGAGTGGTCTGAGAGGAAAGG | 233bp |
| Trapped (KO) allele |  | 58 | F: ATGGCCTGGGATTTGATGACC  R: TGTGAGCGAGTAACAACCCG | 753bp |
| *GFP*  allele | M73708 | 60 | F: CGACGGCAACTACAAGAC  F: TAGTTGTACTCCAGCTTGTGC | 307bp |
| *GFP* (qPCR) | M73708 | 60 | F: CTTTCCCAAGAGAAGGGTCC  R: TGCAGAGACATCTGAATGGC | 107bp |
| *β-Tubulin* | M28739 | 60 | F: GCCAGAGTGGTGCAGGAAATA  R: TCACCACGTCCAGGACAGAGT | 81bp |

**Supplementary Table 2. List of primers used for RT-PCR analysis**

| **Gene** | **Genebank** | **Tm (°C)** | **Primer sequences (5’ to 3’)** | **Product size** |
| --- | --- | --- | --- | --- |
| *Dhh* | NM_007857.5 | 56 | F: GGGACCTCGTACCCAACTAC  R: CTTTGCAACGCTCTGTCATC | 139bp |
| *Shh* | NM_009170.3 | 60 | F: CAGCGACTTCCTCACCTTCCT  R: AGCGTCTCGATCACGTAGAAG | 129bp |
| *Gapdh* | NM_001289726.1 | 60 | F: TGTGTCCGTCGTGGATCTGA  R: CCTGCTTCACCACCTTCTTGA | 77bp |
| *Gli1* | NM_010296.2 | 57 | F: CTATCCTCAGCCTCCCCATG  R: CCTCCCACAACAATTCCTGC | 146bp |
| *Gli2* | NM_001081125.1 | 57 | F: CAGTCCTGAGCTATCCCCAG  R: GAGGCTGCATGAGACCAAAG | 117bp |
| *Gli3* | NM_000168.5 | 57 | F: CTGCAGTGAGAGTGGACAGG  R: GTATCCAGTTGTGGGCTGCT | 162bp |
| *Ptch1* | NM_000264.3 | 55 | F: TGTTCCAGTTAATGACTCCC  R: ACACTCTGATGAACCACCTC | 145bp |
| *Blimp1* | NM_007548.4 | 55 | F: CACACAGGAGAGAAGCCACA  R: TTGTGACACTGGGCACACTT | 209bp |
| *c-Kit* | NM_001122733.1 | 55 | F: TGTAAGGCCTCCAACGATGT  R: ACCACAAAGCCAATGAGCAG | 122bp |
| *Steel* | NM_013598.3 | 55 | F: AGGTCCCGAGAAAGATTCCA  R: TTGTAGGCCCGAGTCTTCAG | 136bp |
| *Cxcr4* | NM_009911.3 | 55 | F: AACCTCTACAGCAGCGTTCT  R: GATCCAGACGCCCACATAGA | 132bp |
| *Sdf-1* | NM_001012477.2 | 55 | F: GCCAACGTCAAGCATCTGAA  R: TTCGGGTCAATGCACACTTG | 101bp |
| *Wdr11* | NM_172255.3 | 60 | F: CATTTGACCAACCACAGCAC  R: GACCACGGACGCTAAACATT | 131bp |
| *Gapdh* | NM_001289726.1 | 60 | F: TGTGTCCGTCGTGGATCTGA  R: CCTGCTTCACCACCTTCTTGA | 170bp |
| *Boc* | NM_172506.2 | 55 | F: TTCATCCCCTTCTGCCTATG  R: ACCATTGTGTACTGGCACGA | 187bp |
| *Cdo* | NM_021339.2 | 55 | F: CCAGTGCGTTGCCAACAACAGC  R:TGGTACCCTGCAGCCAATGAAGC | 148bp |
| *Gas1* | NM_008086.2 | 55 | F: TCAACGACTGCGTGTGCGATGG  R: GGACCGTTGCTCGCATCTGG | 100bp |

**Supplementary Table 3. Guide RNAs used for CRISPR/Cas9**

| **Target gene** | **Targeting sequences (200bp) before PAM** |
| --- | --- |
| *Wdr11*(RC) | F: CACCAAGATAAGCCCCTGGAATTA  R: AAACTAATTCCAGGGGCTTATCTT |
| *Wdr11*(MT/KO) | F: CACCGGATGAACCTTATGAAAGTAG  R: AAACCTACTTTCATAAGGTTCATCC |
| *Ift88* (KO) | F: CACCGGATGAACCTTATGAAAGTAG  R: AAACCTACTTTCATAAGGTTCATCC |

**Supplementary Table 4. ssODN for generating point mutations**

| **Target gene** | **ssODN sequences (90bp)** |
| --- | --- |
| *Wdr11*(RC) | GAAACTTTTAGACATTTCTCTAAGAAGAGTGCAAGTTCTAATATCCCTTAATTCCAGGGGCTTATCTTTGAAAACAACTGCCAAATACTG |
| *Wdr11*(MT) | GAATGGACAAGCTTGACCAGTTTCCTTTCTTTTGCTGCTTCGACCCTAAACAACATGGGGTTAGTAAGAAATGAACTTCAGCTGGTTGAT |

**Supplementary Table 5. Primers used for validation of gene targeting after CRISPR/Cas9**

| **Target gene** | **Primer sequences (5’ to 3’)** |
| --- | --- |
| *Wdr11* (RC) | F: ATGCTGTCTGAGTCCTACCCTC  R: CCACACAGATCAGTACCCAAGA |
| *Wdr11* (MT/KO) | F: TAGGGGTATTGAATGGACAAGC  R: AAACCATGTTGTTTGGGGTCGAAG |
| *Ift88* (KO) | F: TAAGAGTGAACGACTGAGTGCC  R: TAGACAGTGCAAACCCAATGAC |

**SUPPLEMENTARY FIGURE LEGENDS**

**Figure S1. Wdr11 is expressed in PGCs and urogenital tissues.**

**(A)** Genotyping analyses of *Wdr11* knockout mice by PCR. The WT and gene-trap alleles are indicated (left panel). NC, no template control. The presence of the GFP-specific allele in the *Stella^GFP^;Wdr11^+/-^* hybrid line was confirmed by PCR (right panel) as well as by a test breeding (see Materials and Methods).  **(B)** Expression of *Wdr11, Blimp1, c-Kit (Kit), Steel (Kitl), Cxcr4, Sdf1* and *Gapdh* in the hindgut (HG) at E9.5, the urogenital ridge area (UG) at E10.5 - 11.5 and the post-pubertal reproductive organs (testis, epididymis, ovary) and kidney in 8-week-old wild-type mice was assessed by RT-PCR. Representative results are shown from 3 independent biological samples. NC, no template control. **(C)** Images of transverse sections of whole-mount X-gal-stained paraffin-embedded E10.5 embryos with eosin counterstaining. WT (+/+) and heterozygous (+/-) embryos are shown (scale bar, 100 μm). n, neural tube; ao, aorta; nc, notochord; mt, mesonephric tubules; hg, hindgut. **(D)** Transverse sections of E10.5 *Wdr11* WT and KO embryos stained with anti-WDR11 (green) and anti-SSEA1 (red) antibodies and DAPI (blue), confirming the expression of WDR11 in PGCs (SSEA1-positive) and the surrounding somatic cells in the GR area. A zoomed-in image of the dotted area is shown. The scale bar represents 100 μm and 20 μm (zoomed-in image).

**Figure S2. Primary cilia are disrupted in the Wdr11-null PGC migratory niche.**

**(A)** A representative immunofluorescence image of 3D reconstruction of GR region in E10.5 WT embryo stained with anti-Arl13B (green) and anti-SSEA1 (red) antibodies and DAPI (blue). ImageJ software using the volume viewer plugin was used to build the image stacks. Scale bar, 10μm. **(B)** Analyses of primary cilia on WT and Wdr11^-/-^ GR sections. Zoomed-in images of the dotted area are shown on the right. Scale bar, 10μm (left panels) and 50μm (right panels). Representative images are shown from 3 independent biological samples. **(C)** Comparison of the ciliation frequency and cilium length in GR sections. Ciliation frequency values generated from the total number of cilia and nuclei counted from the maximum intensity projection images of each channel manually. Cilia length assessed by measuring the maximum projection of Arl13b signal using ImageJ. WT (n=119) and *Wdr11*^-/-^ (n=103). Unpaired t-test. ***P<0.001. **(D)** Transmission electron micrographs (15000x) of sections of 8-week old brain tissue. Scale bars, 600nm. The mother centriole (basal body), daughter centriole and cilia appendages are recognisable as indicated.

**Figure S3. WDR11-deficient mice show dysgenesis of gonads.**

**(A, B)** Hematoxylin and eosin-stained sections of WT and *Wdr11^-/-^* mouse ovaries are shown. *Wdr11^-/-^* ovaries are smaller compared to WT and contain a markedly reduced number of oocytes and mature follicles, with disproportionally higher numbers of oogonia or primordial follicles, showing arrested follicle development as indicated by the absence of corpora lutea and antral follicles. Note, the data for *Wdr11^-/-^* male mouse testes and sperms have been previously reported (Kim et al., 2018).

**Figure S4. Loss of WDR11 does not influence cell death and survival.**

**(A)** Representative images of immunofluorescence staining of SSEA1 (red) and Caspase-3 (CASP3, green) on E10.5 WT and *Wdr11^-/-^* embryos. The yellow line indicates the GRs. Scale bar, 100 µm. **(B)** Analyses of apoptosis based on Caspase-3 immunofluorescence. CASP3-positive and total cells were counted from every other section of the PGC migratory route (top left). The percentages of CASP3-positive somatic cells (top right), PGCs (bottom left) and ectopic PGCs (bottom right) are compared between WT and KO embryos. Error bars represent SEM. Statistical analysis by unpaired Student's t-test (n=5, number of embryos for each genotype; ***P<0.001).

**Figure S5. Expression profile of PGC regulatory genes.**

Comparison of expression levels of Blimp1, c-Kit, Steel, Cxcr4 and Sdf1 in WT and *Wdr11^-/-^* GR tissues by quantitative RT-PCR. Loss of WDR11 did not affect the expression of these genes in the PGC migratory niche area, except c-kit which showed a small but statistically significant decrease. Values are shown as means ± SD. Unpaired Student's t-test (n=5 embryos for each genotype; **P < 0.01).

**Figure S6. Characterisation of NIH3T3 cells after CRISPR/Cas9-mediated mutagenesis.**

**(A)** Western blot analyses of NIH3T3/Cas9 cells with targeted gene editing confirmed the absence of endogenous proteins after KO. The missense variants Wdr11-RC and Wdr11-MT still produced the full-length proteins although at reduced levels. All variants were validated by direct Sanger sequencing of genomic DNA. **(B)** The phase contrast microscope imaging (scale bar 100 μm) and F-actin phalloidin staining (scale bar 100 μm) showed that the gross cell morphology and cytoskeletal organisation of different NIH3T3/Cas9 cells were not altered significantly. **(C)** The primary cilium (scale bar 10 μm) was visualised with Arl13B labelling for axoneme (green) and gamma-tubulin labelling for the basal body (red). Representative images are shown. Quantitative analyses of the cilia length are shown in Figure S7B.

**Figure S7.** **Characterisation of NIH3T3/Cas9 cells with WDR11 mutations.**

**(A)** Growth curves of NIH3T3/Cas9 expressing different mutations. Cells plated at 2x10^6^ per 10 cm dish in the normal growth medium were counted at the time points indicated. Values are means ± SD. **(B)** NIH3T3/Cas9 cells plated onto the glass cover slips coated with 0.001% poly-L-lysine in PBS were incubated in serum-free medium for 24 hours to induce primary cilia formation. The length of the cilia axoneme in the random fields of cells measured from WT (n=186), Wdr11-RC (n=98), Wdr11-MT (n=52), Wdr11 KO (n=101) and Ift88 KO (n=68) cells. Values are means ± SD after an unpaired t-test with Welch’s correction. *P<0.01, ****P<0.0001.

**Figure S8. Loss of WDR11 disrupts HH pathway gene expression in GR tissues.**

**(A)** Expression of Ptch1, Gli1, Gli2 and Gli3 in the PGC migratory niche of WT mouse embryos assessed by RT-qPCR normalised to Gapdh at the stages indicated. Means ± SD is shown after multiple t-test (n=5 per stage). *P<0.01, **P<0.001; ***P<0.0001; *****P<0.000001. **(B)** Expression levels of Ptch1, Gli1, Gli2 and Gli3 in the GR area of WT and *Wdr11^-/-^* embryos. Means ± SD are shown with t-test (n=5 per genotype); **P = 0.003730; ***P=0.000045. **(C)** Boc mRNA levels in the GR area of WT and Wdr11^-/-^ embryos were assessed by RT-qPCR. Values are means ± SD after the t-test (n=5 per genotype). **(D)** Dhh mRNA levels in the E9.5 – E12.5 GR region did not show a significant difference by Welch’s ANOVA test (n=5 embryos). **(E)** Expression of Dhh and Shh in the GRs did not show significant difference by t-test (n=5 per genotype); P = 0.76, P = 0.29, respectively.

**Figure S9. Defective noncanonical HH signalling in** ***Wdr11***^-/-^ **PGCs.**

**(A)** Additional images for immunofluorescence analyses of p-Creb, supplementing Figure 5C. **(B)** Representative merged images of immunofluorescence staining of SSEA1 (red) and Gli3 (green) shown with DAPI (blue) on E10.5 WT and *Wdr11^-/-^* embryos. Gli3 expression is barely detectable in PGCs with or without Shh-N stimulation. Scale bar, 100 µm.

**SUPPLEMENTARY MOVIE LEGENDS**

**Supplementary Movie 1. PGC migration in E10.5 WT embryo.**

A time-lapse movie of an embryo slice culture from *Stella^GFP+/+^;Wdr11^+/+^* mouse as described in the Materials and Methods. PGCs are labelled as green fluorescence.

**Supplementary Movie 2. PGC migration in E10.5 Wdr11^-/-^ embryo.**

A time-lapse movie of an embryo slice culture from *Stella^GFP+/+^;Wdr11^-/-^* mouse as described in the Materials and Methods. PGCs are labelled as green fluorescence.

**Supplementary Movie 3. WT genital ridge co-culture on WT feeder.**

A time-lapse movie of E10.5 *Stella^GFP+/+^* mouse GR cells cultured on WT NIH3T3/Cas9 cell feeder.

**Supplementary Movie 4. WT genital ridge co-culture on Wdr11-RC feeder.**

A time-lapse movie of E10.5 *Stella^GFP+/+^* mouse GR cells cultured on NIH3T3/Cas9 cell feeder edited for Wdr11-RC mutation.

**Supplementary Movie 5. WT genital ridge co-culture on Wdr11-MT feeder.**

A time-lapse movie of E10.5 *Stella^GFP+/+^* mouse GR cells cultured on NIH3T3/Cas9 cell feeder edited for Wdr11-MT mutation.

**Supplementary Movie 6. WT genital ridge co-culture on Wdr11 KO feeder.**

A time-lapse movie of E10.5 *Stella^GFP+/+^* mouse GR cells cultured on NIH3T3/Cas9 cell feeder with Wdr11 KO.

**Supplementary Movie 7. WT genital ridge co-culture on IFT88 KO feeder.**

A time-lapse movie of E10.5 *Stella^GFP+/+^* mouse GR cells cultured on NIH3T3/Cas9 cell feeder with Ift88 KO.

**Supplementary Movie 8. WT genital ridge co-culture on WT feeder treated with DMF.**

A time-lapse movie of E10.5 *Stella^GFP+/+^* mouse GR cells cultured on WT NIH3T3/Cas9 cell feeder after treatment with DMF.

**Supplementary Movie 9. WT genital ridge co-culture on WT feeder treated with Shh-N.**

A time-lapse movie of E10.5 *Stella^GFP+/+^* mouse GR cells cultured on WT NIH3T3/Cas9 cell feeder after treatment with Shh-N.

**Supplementary Movie 10. WT genital ridge co-culture on Wdr11 KO feeder treated with DMF.**

A time-lapse movie of E10.5 *Stella^GFP+/+^* mouse GR cells cultured on NIH3T3/Cas9 cell feeder with Wdr11 KO after treatment with DMF.

**Supplementary Movie 11. WT genital ridge co-culture on Wdr11 KO feeder treated with Shh-N.**

A time-lapse movie of E10.5 *Stella^GFP+/+^* mouse GR cells cultured on NIH3T3/Cas9 cell feeder with Wdr11 KO after treatment with Shh-N.
