# Supplementary material for: Coordination of canonical and noncanonical Hedgehog signalling pathways mediated by WDR11 during primordial germ cell development
Source: Sci Rep. 2023 Jul 29;13:12309. doi: 10.1038/s41598-023-38017-9 (PMC10387110; doi:10.1038/s41598-023-38017-9)
Supplement: Supplementary file 3 — Supplementary Figures. [file 41598_2023_38017_MOESM3_ESM.pptx]

## Slide 1
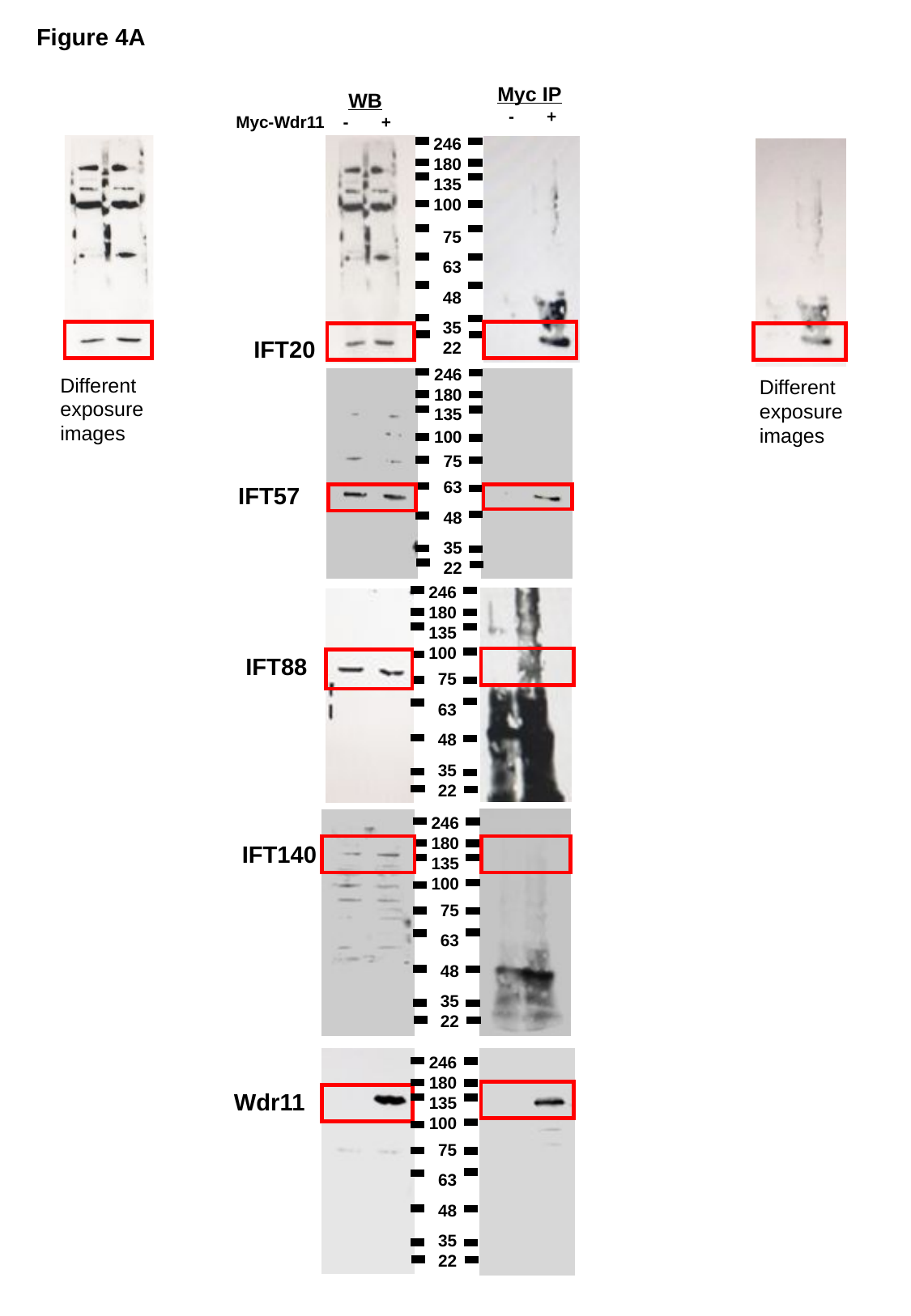

Figure 4A
Myc IP
WB
 - +
Myc-Wdr11 - +
246
180
135
100
75
63
48
35
22
IFT20
246
180
135
100
75
63
48
35
22
Different exposure images
Different exposure images
IFT57
246
180
135
100
75
63
48
35
22
IFT88
246
180
135
100
75
63
48
35
22
IFT140
246
180
135
100
75
63
48
35
22
Wdr11

## Slide 2
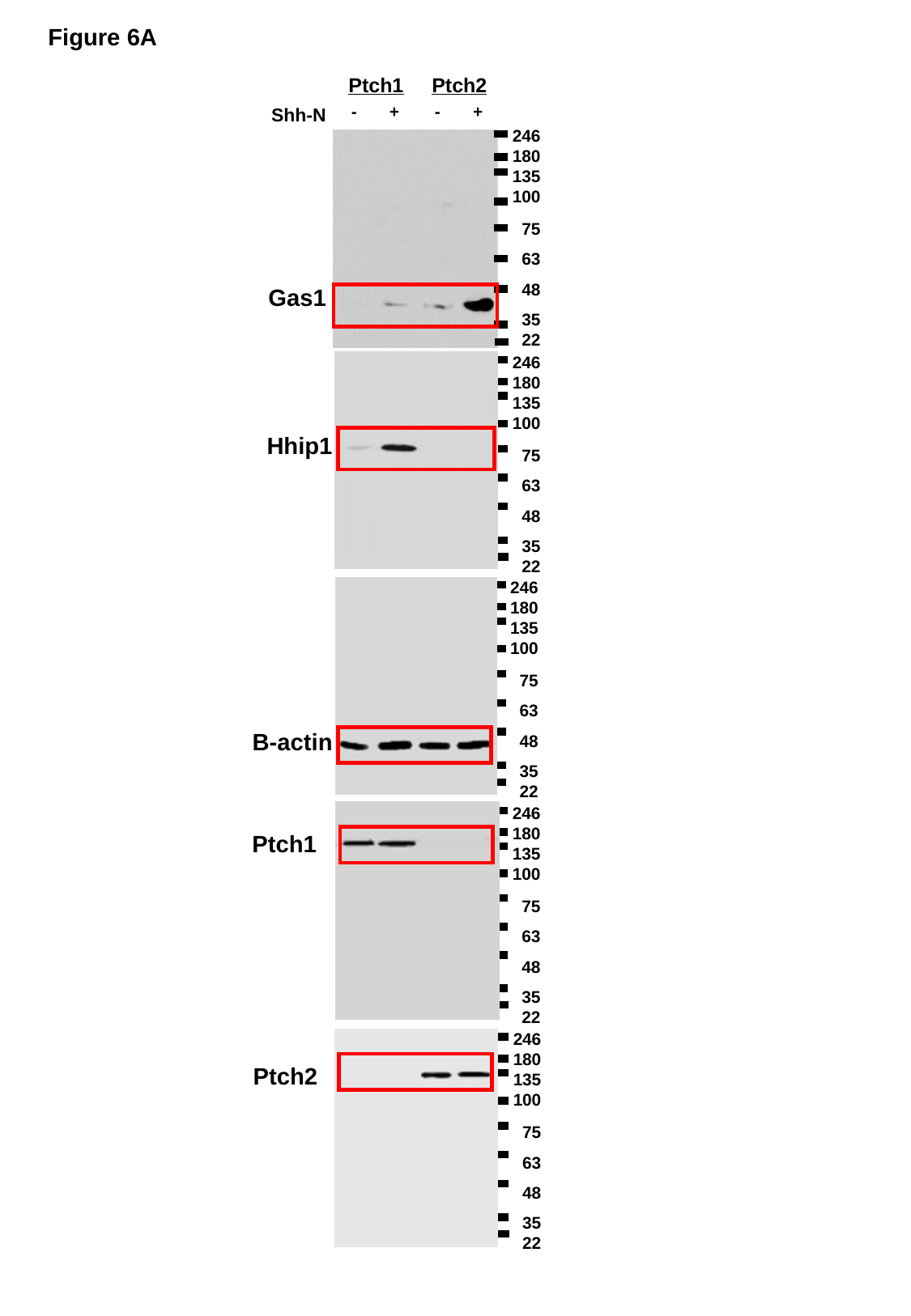

Figure 6A
Ptch1
Ptch2
- +
- +
246
180
135
100
75
63
48
35
22
Gas1
246
180
135
100
75
63
48
35
22
Hhip1
246
180
135
100
75
63
48
35
22
B-actin
246
180
135
100
75
63
48
35
22
Ptch1
246
180
135
100
75
63
48
35
22
Ptch2
Shh-N

## Slide 3
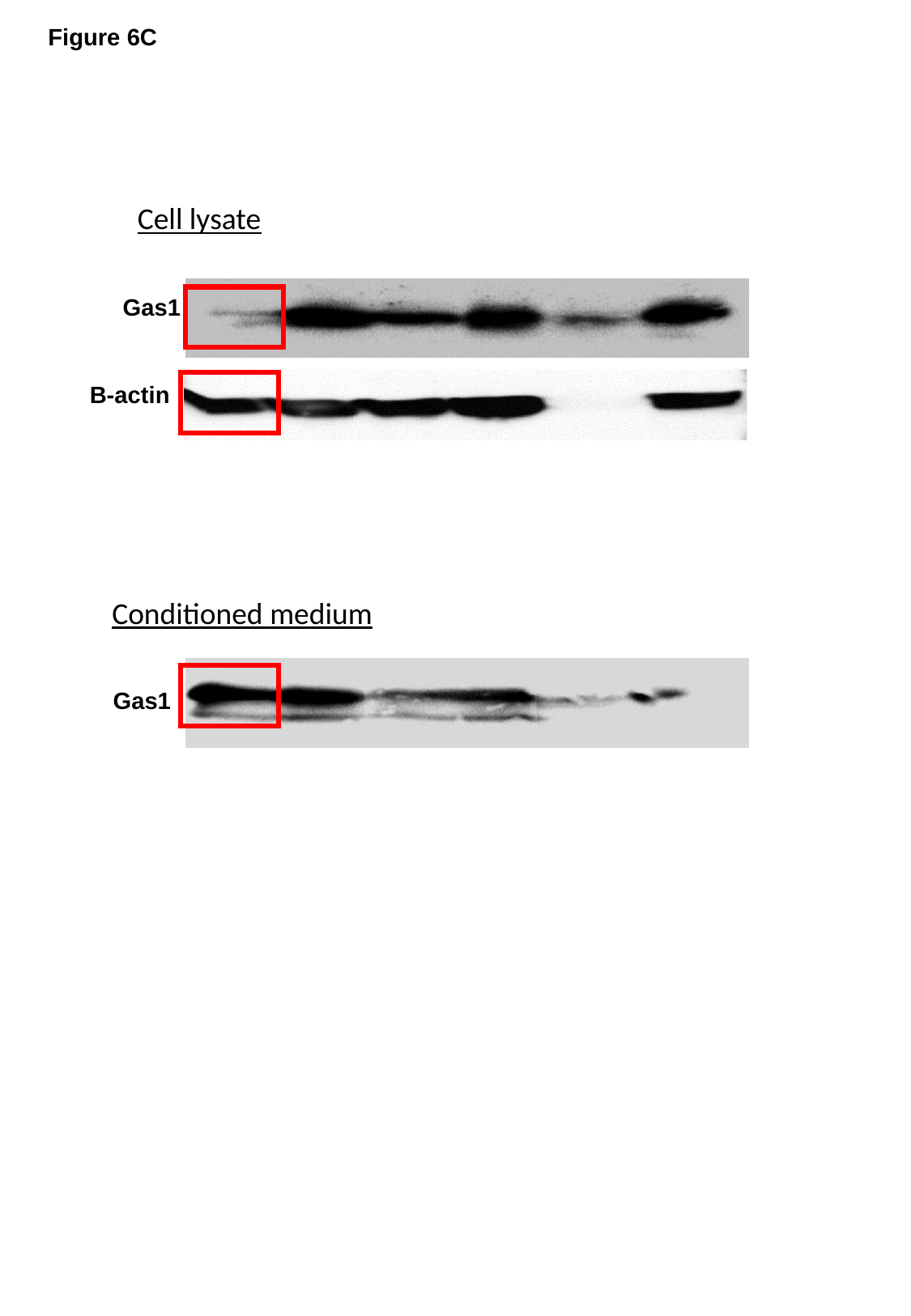

Figure 6C
Cell lysate
Gas1
B-actin
Conditioned medium
Gas1

## Slide 4
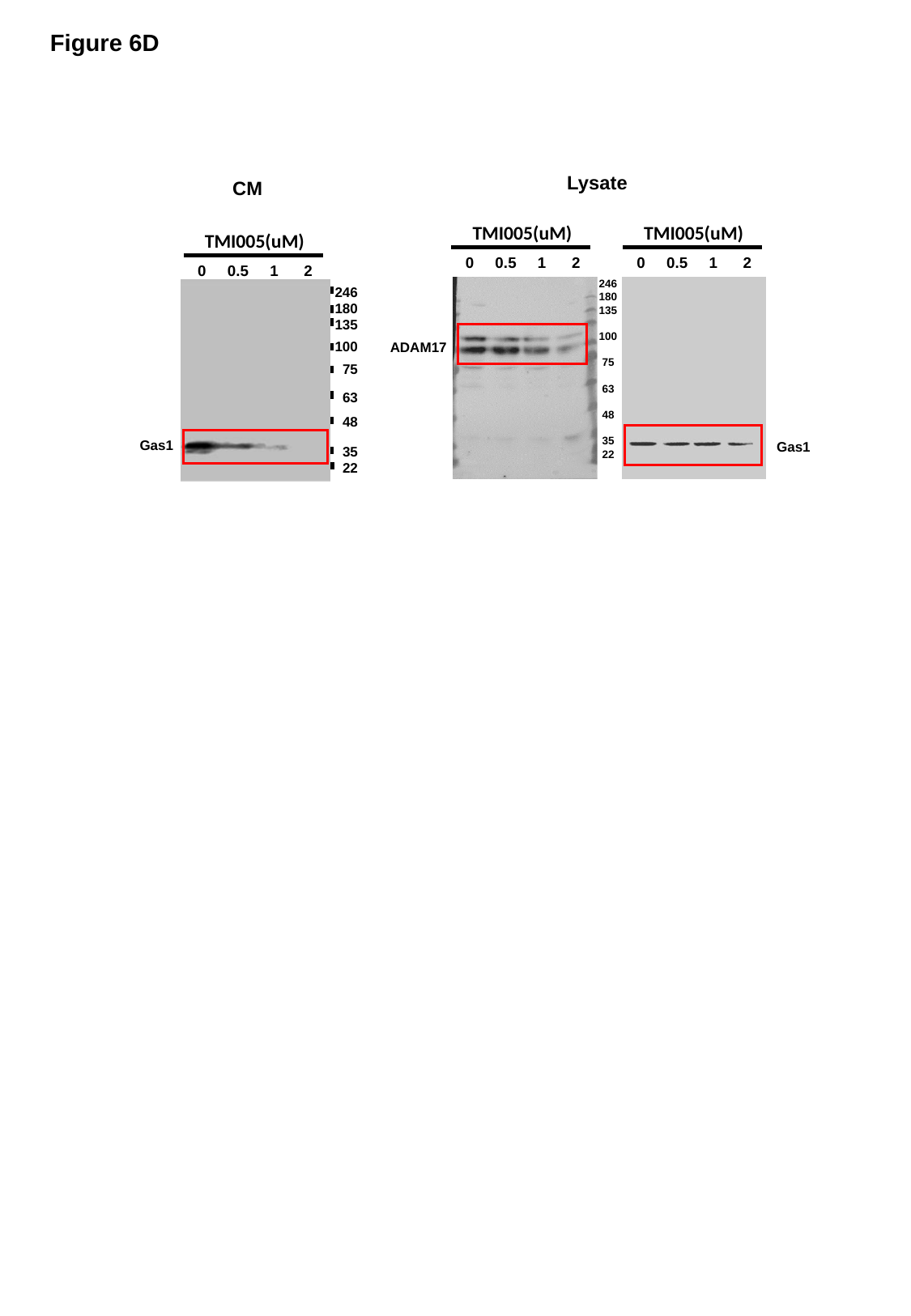

Figure 6D
Lysate
CM
TMI005(uM)
TMI005(uM)
TMI005(uM)
0 0.5 1 2
0 0.5 1 2
0 0.5 1 2
246
180
135
100
75
63
48
35
22
ADAM17
Gas1
Gas1
246
180
135
100
75
63
48
35
22

## Slide 5
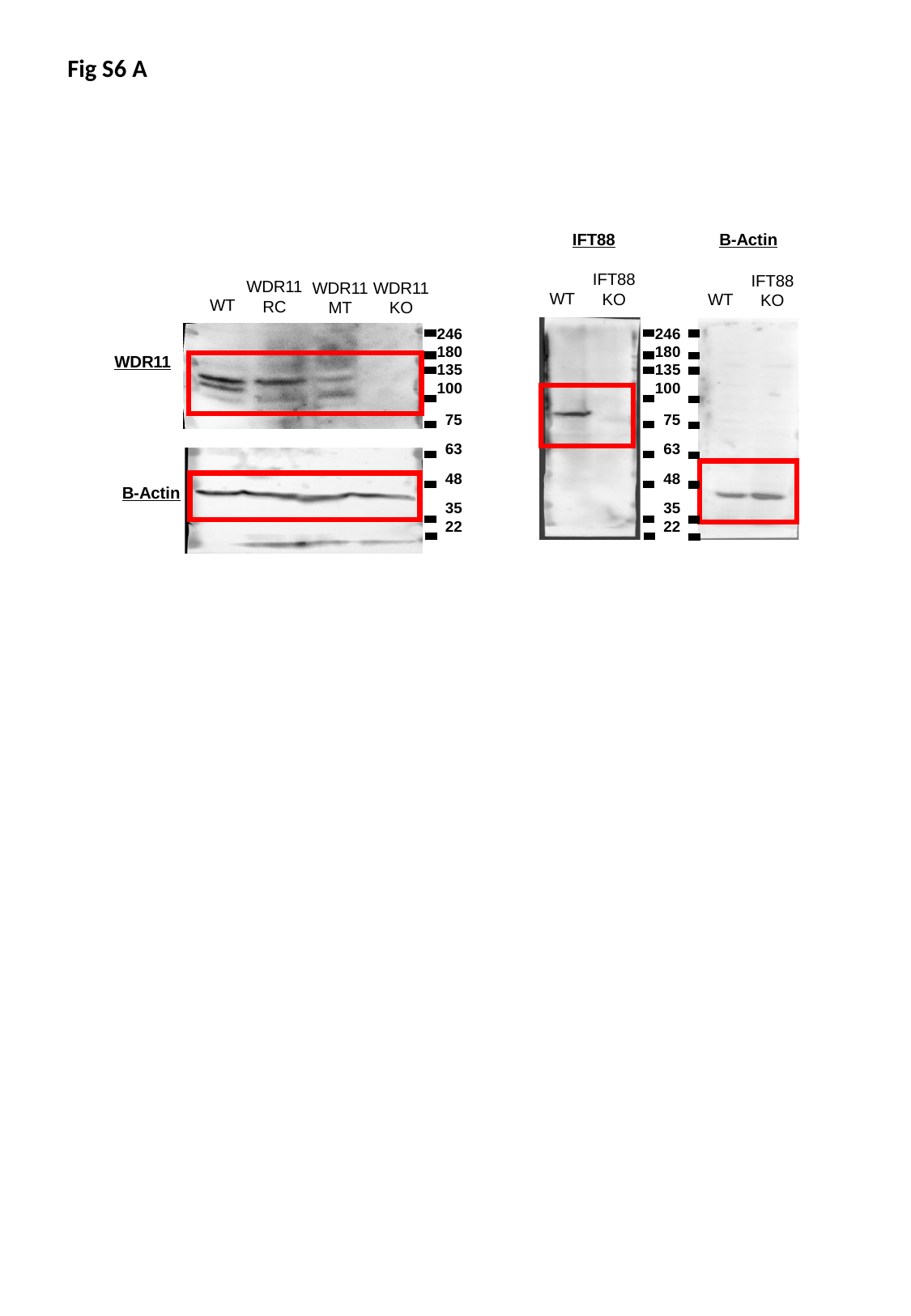

Fig S6 A
IFT88
B-Actin
IFT88
KO
IFT88
KO
WDR11
RC
WDR11
MT
WDR11
KO
WT
WT
WT
246
180
135
100
75
63
48
35
22
246
180
135
100
75
63
48
35
22
WDR11
B-Actin
